# Cohesin Causes Replicative DNA Damage by Trapping DNA Topological Stress

**DOI:** 10.1016/j.molcel.2020.03.013

**Published:** 2020-05-21

**Authors:** Nicola Elizabeth Minchell, Andrea Keszthelyi, Jonathan Baxter

**Affiliations:** 1 Genome Damage and Stability Centre, School of Life Sciences, Science Park Road, University of Sussex, Falmer, Brighton, East Sussex BN1 9RQ, UK

**Keywords:** DNA replication stress, DNA topology, topoisomerases, SMC, cohesin, condensin, DNA damage

## Abstract

DNA topological stress inhibits DNA replication fork (RF) progression and contributes to DNA replication stress. In *Saccharomyces cerevisiae*, we demonstrate that centromeric DNA and the rDNA array are especially vulnerable to DNA topological stress during replication. The activity of the SMC complexes cohesin and condensin are linked to both the generation and repair of DNA topological-stress-linked damage in these regions. At cohesin-enriched centromeres, cohesin activity causes the accumulation of DNA damage, RF rotation, and pre-catenation, confirming that cohesin-dependent DNA topological stress impacts on normal replication progression. In contrast, at the rDNA, cohesin and condensin activity inhibit the repair of damage caused by DNA topological stress. We propose that, as well as generally acting to ensure faithful genetic inheritance, SMCs can disrupt genome stability by trapping DNA topological stress.

## Highlights

• DNA topological stress causes DNA damage during DNA replication
• Cohesin activity causes topological-stress-dependent DNA damage at centromeres
• Cohesin and condensin activities delay the repair of DNA damage across the rDNA
• Chromosomal cohesin causes the accumulation of DNA topological stress on plasmids

## Graphical Abstract

**Figure:**
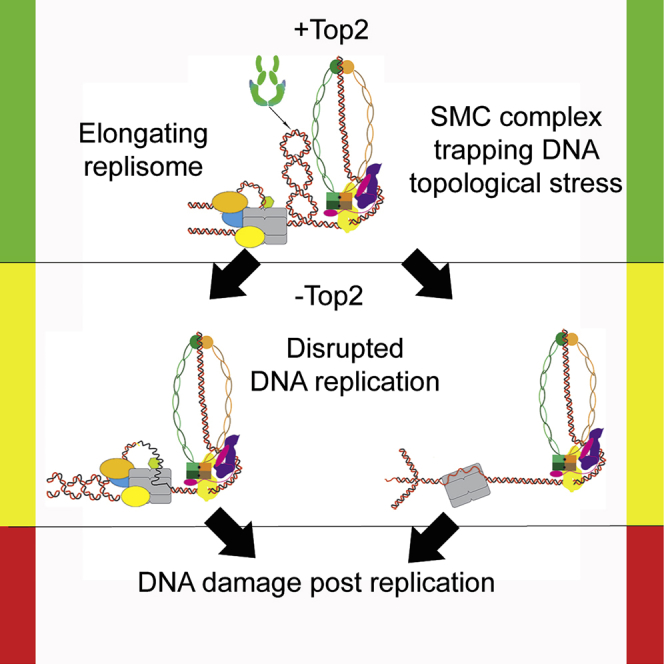

## Teaser

Minchell et al. show that the SMC complex cohesin causes DNA topological stress in chromosomes during DNA replication. Topoisomerase II activity is required during S phase to resolve cohesin-dependent DNA topological stress accumulating at centromeres and prevent the disruption of DNA replication causing DNA damage.

## Introduction

Chronic DNA replication stress is a widespread source of DNA damage in cancerous cells (Macheret and Halazonetis, 2015). DNA damage related to DNA replication is focused within genomic contexts that challenge normal DNA replication fork (RF) progression (Aguilera and García-Muse, 2013). These include DNA sequences that form secondary structures or regions that stably bind protein complexes (Ivessa et al., 2003, Mirkin and Mirkin, 2007). In addition, the action of the replicative helicase itself generates a countervailing impediment to RF progression (Keszthelyi et al., 2016). The separation of the two DNA strands by the helicase displaces the intertwining between them into the region ahead of the RF. This increases the frequency of linkages between the strands above the ideal winding density for relaxed DNA, generating DNA topological stress (Postow et al., 2001a). If allowed to accumulate, DNA topological stress ahead of the RF will inhibit unwinding and impede elongation (Bermejo et al., 2007, Brill et al., 1987, Keszthelyi et al., 2016). This is normally prevented either by the rapid action of cellular topoisomerases or by diffusion of the topological stress through the duplex DNA, minimizing the possibility of the acute topological stress stalling RF progression while maximizing the substrate available for topoisomerase action (Postow et al., 2001a). Acute accumulation of topological stress is proposed to occur when the wave of DNA topological stress generated ahead of the RF converges with a similar wave of overwound topological stress ahead of transcribing RNA polymerase complexes (Bermejo et al., 2011, Olavarrieta et al., 2002). Alternatively, DNA topological stress is also thought to accumulate at structures predicted to impede free rotation of DNA (Bermejo et al., 2011, Schalbetter et al., 2015). These potentially lead to rates of local stress accumulation that exceed the relaxation activity of cellular topoisomerases (Schalbetter et al., 2015). The consequences of impeding the RF through elevated topological stress can include fork reversal, to stabilize the arrested RF, or fork rotation, which promotes transformation of overwinding stress ahead of the RF into pre-catenanes behind the fork, allowing further elongation without topoisomerase action ahead of the RF (Keszthelyi et al., 2016, Peter et al., 1998, Postow et al., 2001b, Schalbetter et al., 2015). At present, the chromosomal regions that actually accumulate sufficiently high levels of topological stress to impede RF progression and cause replication stress and DNA damage are unknown.

Because DNA topological stress is an endogenous cause of DNA damage, sites that are preferentially vulnerable to DNA topological stress during DNA replication should be within the set of genomic regions known to accumulate DNA damage in the absence of exogenous agents. Known sites of endogenous DNA damage in budding yeast, identified by local enrichment of γH2AX (H2AS129P in *Saccharomyces cerevisiae*), include a number of sites known to impede the RF, including centromeres and the rDNA repeats (Szilard et al., 2010).

In eukaryotes, the three SMC complexes cohesin, condensin, and SMC5/6 all have distinct roles in ensuring faithful chromosomal inheritance in cycling cells (Uhlmann, 2016). SMC complexes translocate along DNA fibers (Ganji et al., 2018, Terakawa et al., 2017), either generating *cis* loops along DNA (Gibcus et al., 2018, Nasmyth, 2001, Rao et al., 2017, Schalbetter et al., 2017, Schwarzer et al., 2017) or connecting sister chromatids (Haering et al., 2008). SMCs also promote genome stability following replication stress. Cohesin action during S phase promotes fork stability following replication stress (Frattini et al., 2017, Fumasoni et al., 2015) and facilitates double strand break repair (Sjögren and Nasmyth, 2001, Ström et al., 2007, Unal et al., 2007). SMC5/6 facilitates DNA repair after replication stress and DNA strand breakage (Aragón, 2018).

To establish which genomic contexts impede DNA replication through accumulation of DNA topological stress, we have analyzed where DNA damage accumulates following DNA replication in cells depleted of Topoisomerase II (Top2) and examined the effects of defined chromosomal features on replication-dependent topological stress on plasmids. Surprisingly, we find that activity of cohesin during S phase comes at the cost of generating additional DNA topological stress on chromosomes, leading to endogenous DNA damage around centromeres, which is minimized by the activity of Top2 during DNA replication.

## Results

**Figure 1:**
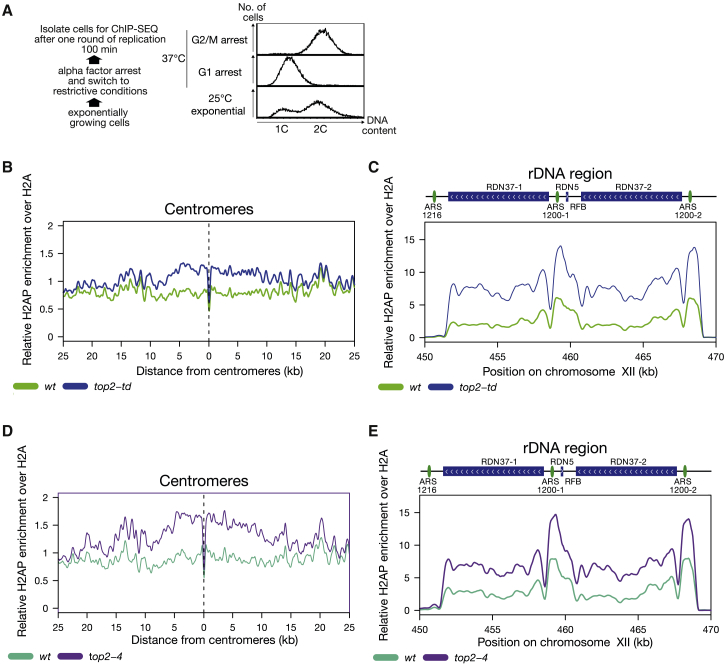
Depletion of Top2 during S Phase Causes H2AS129P Enrichment at Centromeres and over the rDNA Repeats (A) Experimental setup of ChIP-seq experiments, indicating how the post-replication cell populations used for the ChIP-seq experiments were prepared. A representative FACS analysis of DNA content of each of the indicated stages of the experiment is shown. (B) The relative enrichment of H2AS129P over H2A ChIP around centromeres in cells is shown either with wild-type expression of Top2 in parental cells (green) or depleted of Top2 (blue) in *top2-td* cells, both released into the cell cycle under the restrictive conditions. Graph shown is generated from a pile up of the profiles of all centromeres and is an average of two repeats. (C) The relative enrichment of H2AS129P over H2A ChIP across the rDNA repeats in cells either with wild-type expression of Top2 (green) or depleted of Top2 (blue) in *top2-td* cells, both released into the cell cycle under the restrictive conditions. Graph shown is an average of two repeats. (D) The relative enrichment of H2AS129P over H2A ChIP around centromeres in cells is shown either with wild-type expression of Top2 in parental cells (turquoise) or in *top2-4* cells (purple), both released into the cell cycle under the restrictive conditions. Graph shown is generated from a pile up of the profiles of all centromeres and is an average of two repeats. (E) The relative enrichment of H2AS129P over H2A ChIP across the rDNA repeats in cells either with wild-type expression of Top2 in parental cells (turquoise) or in *top2-4* cells (purple), both released into the cell cycle under the restrictive conditions. Graph shown is an average of two repeats. See also Figures S1, S2, and S7.

To identify chromosomal contexts where DNA topological stress leads to DNA-replication-associated damage, we examined cells depleted of Top2 (Baxter and Diffley, 2008) during S phase. Due to the presence of Top1, depletion of Top2 does not prevent bulk DNA replication or lead to pre-mitotic cell cycle arrest (Baxter and Diffley, 2008, Bermejo et al., 2007, Holm et al., 1985). However, we have previously observed an increase in cellular H2AS129P during S phase in cells where Top2 was rapidly degraded using the *top2-td* allele prior to replication (Schalbetter et al., 2015), suggesting a subset of RFs are disrupted by the increase in topological stress occurring in Top2-depleted cells. In order to identify the regions where RF progression is particularly vulnerable to DNA topological stress, we carried out H2AS129P chromatin immunoprecipitation, followed by next generation sequencing of immunoprecipitated DNA (ChIP-seq) following DNA replication. We arrested parental or *top2-td* degron cells in G1 with alpha factor before incubation at the restrictive conditions to deplete Top2. We released the cells into the cell cycle by alpha factor wash-out, allowing them to complete a single S phase (Figure 1A) and taking the cells for analysis 100 min after release from alpha factor. DNA damage due to passage through mitosis was prevented by incubating the cells with the microtubule depolymerizing drug nocodazole. We then used ChIP-seq to identify chromosomal regions where H2AS129P was elevated relative to H2A in parental and Top2-depleted cells. We observed two genomic contexts where H2AS129P was consistently increased in Top2-depleted cells, around the centromeric regions and across the rDNA array (Figures 1B and 1C). H2AS129P was increased around all centromeres extending 10–20 kb either side of the kinetochore (Figure S1A). Centromeres connected to long chromosome arms (>250 kb) accumulated more DNA damage than those connected to short chromosome arms (<250 kb; Figure S1B), consistent with proximity to telomeres lowering DNA topological stress in associated regions due to stress diffusion (Joshi et al., 2010). To confirm that the increase in DNA damage was not related to our method of depleting Top2, we repeated the experiments using the extensively characterized *top2-4* allele (Holm et al., 1985). Incubation of the cells containing the *top2-4* allele at the restrictive temperature specifically through S phase also led to high levels of H2AS129P across centromeres and over the rDNA (Figures 1D and 1E).

Both centromeres and the rDNA have previously been linked to genome instability related to loss of Top2 function. Partial loss of Top2 function causes extensive sister chromatid exchange of the rDNA repeats (Christman et al., 1988), indicating that these sequences are especially sensitive to DNA topological stress. Also, several yeast centromeres have been found to be within the proximity of DNA replication termination zones (TERs) (Fachinetti et al., 2010). At TERs, loss of Top2 delays fork convergence and leads to the accumulation of DNA damage around TER sites following cell division (Fachinetti et al., 2010). To establish whether the DNA damage we observed prior to mitosis was related to a role of Top2 in termination, we examined the change in H2AS129P at the characterized TERs (Figure S2). We found that, although we observed an increase in H2AS129P around TER zones, this was entirely due to the presence of the centromeric sites in this set (Figure S2A). Conversely, if we removed data 10 kb either side of TERs from our analysis of the centromeric regions, we still observed an increase in H2AS129P around centromeres following DNA replication (Figure S2B). We conclude that the high levels of H2AS129P around centromeres following S phase are specific to centromeres and not due to being in the locality of termination zones.

**Figure 2:**
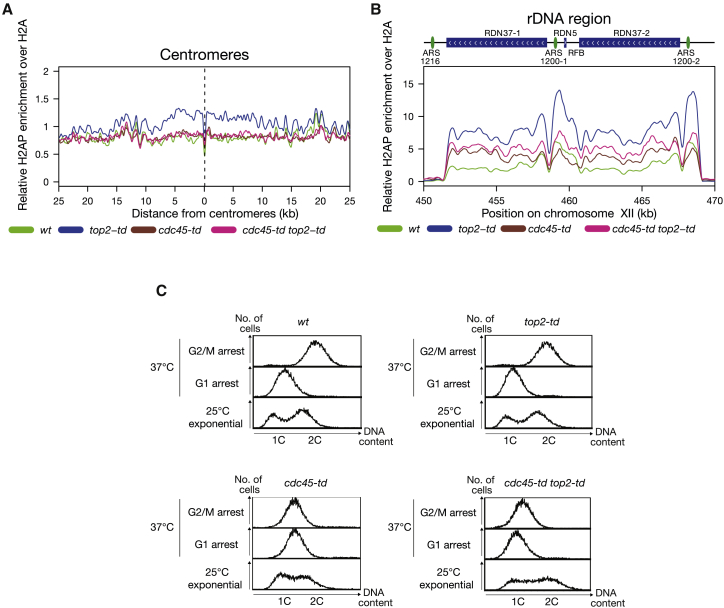
Depletion of Top2 during S Phase Causes H2AS129P Enrichment at Centromeres and over the rDNA Repeats in a Replication-Fork-Dependent Manner (A) The relative enrichment of H2AS129P over H2A ChIP around all centromeres in *cdc45-td*-depleted cells either with WT expression of Top2 in *cdc45-td* (brown) or depleted for Top2 and Cdc45 (pink) in *cdc45-td top2-td* cells, both released into the cell cycle under the restrictive conditions. Data for WT (green) and *top2-td* cells (blue) from Figure 1 are shown for comparison. Graph shown is generated from a pile up of the profiles of all centromeres and is an average of two repeats. (B) The relative enrichment of H2AS129P over H2A ChIP across the rDNA repeats in *cdc45-td*-depleted cells either with WT expression of Top2 in *cdc45-td* (brown) or depleted for Top2 and Cdc45 (pink) in *cdc45-td top2-td* cells, both released into the cell cycle under the restrictive conditions. Data for WT (green) and *top2-td* cells (blue) from Figure 1 are shown for comparison. Graph shown is an average of two repeats. (C) FACS analysis of DNA content for one repeat of each of the indicated stages of the experiment. Second repeat is shown in Figure S7.

In order to confirm that the increase in H2AS129P observed in Top2-depleted cells was due to passage of RFs through these regions rather than cell cycle arrest, we examined H2AS129P enrichment in cells lacking the replication factor Cdc45. Cells depleted of Cdc45 in G1 pass through S and into M phase without DNA replication (Tercero et al., 2000). Inhibition of DNA replication suppressed the increase in H2AS129P across centromeres in Top2-depleted cells (Figure 2A). Across the rDNA, we surprisingly observed an overall increase in H2AS129P in cells depleted of Cdc45 alone (Figure 2B), compared to normal replicating cells, despite not being able to detect any DNA replication in these cells by fluorescence-activated cell sorting (FACS) (Figure 2C). This indicates that RF passage through the rDNA is required to minimize post-replicative DNA damage across the rDNA repeats (Figure 2B). Crucially, the additional loss of Top2 did not increase the level of H2AS129P across the rDNA in Cdc45-depleted cells to the extent observed in replicating cells (Figure 2B). We conclude that the increase in H2AS129P across centromeres and the rDNA in Top2-depleted cells is dependent on the passage of DNA RFs.

**Figure 3:**
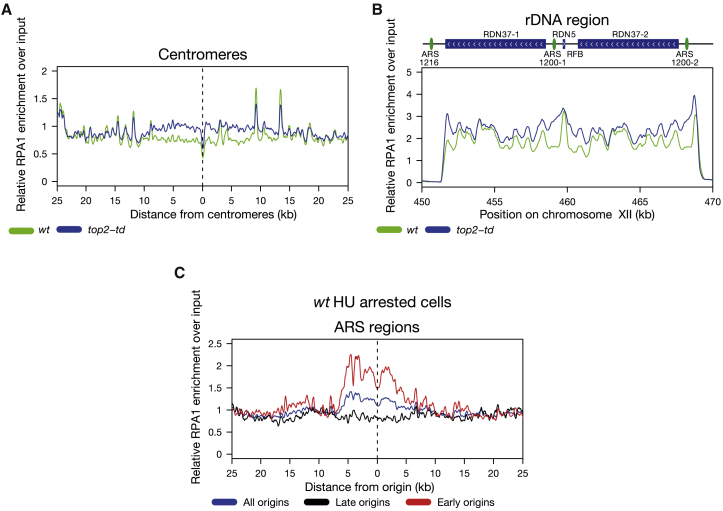
Depletion of Top2 during S Phase Causes H2AS129P Enrichment at Centromeres and over the rDNA Repeats in a Manner that Causes Accumulation of ssDNA (A) The relative enrichment of RPA1-ChIP over input around all centromeres in cells either with WT expression of Top2 in parental cells (green) or depleted of Top2 (blue) in *top2-td* cells, both released into the cell cycle under the restrictive conditions. Graph shown is generated from a pile up of the profiles of all centromeres and is an average of three repeats. (B) The relative enrichment of RPA1-ChIP over input across the rDNA repeats in cells either with WT expression of Top2 (green) or depleted of Top2 (blue), both released into the cell cycle under the restrictive conditions. Graph shown is an average of three repeats. (C) The relative enrichment of RPA1-ChIP over input in WT cells released into 200 mM HU under the restrictive conditions for 60 min around all origins (blue), late origins (black), and early origins (red). Origin data were used from Soriano et al. (2014). Graph shown represents one experiment. See also Figure S7.

Mec1/ATR activation, which is required to generate H2AS129P/γH2AX, is triggered either by the presence of single-stranded DNA (ssDNA) at stalled RFs (Zou and Elledge, 2003) or through local mechanical stress introducing tension into chromosomes (Kumar et al., 2014). To differentiate between these two possibilities, we assayed the accumulation of the ssDNA-binding protein RPA1 by ChIP-seq in replicated cells with and without Top2. We observed increased RPA1 chromatin binding across both the centromeres and rDNA in Top2-depleted cells compared to parental cells (Figures 3A and 3B). Global stalling of DNA replication by hydroxyurea (HU) results in accumulation of H2AS129P around all stalled forks in addition to robust activation of checkpoint effector kinases and pre-mitotic arrest (Puddu et al., 2011). Comparison of the increase in RPA1 across centromeres and the rDNA to the levels of RPA1 accumulation observed around early origins following complete global replication stalling was consistent with only a subset of RFs arresting in these regions in response to topological stress (Figure 3C), resulting in stochastic local accumulation of ssDNA and activation of Mec1^ATR^.

**Figure 4:**
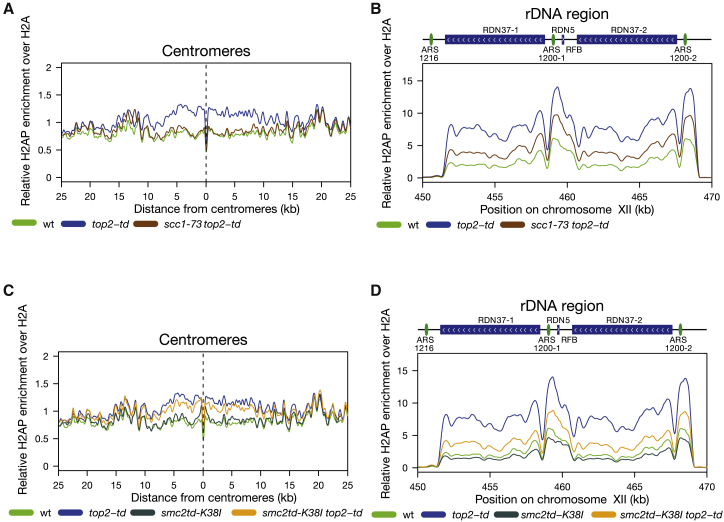
Inactivation of Cohesin Suppresses H2AS129P Enrichment around Centromeres, although Inactivation of Cohesin or Condensin Suppresses H2AS129P Enrichment across the rDNA Repeats (A) The relative enrichment of H2AS129P over H2A ChIP around centromeres is shown for *scc1-73 top2-td* (brown), released into the cell cycle under the restrictive conditions. Data for WT parental cells (green) and *top2-td* cells (blue) from Figure 1 are shown for comparison. Graph shown is generated from a pile up of the profiles of all centromeres and is an average of two repeats. (B) The relative enrichment of H2AS129P over H2A ChIP over the rDNA repeats is shown for *scc1-73 top2-td* (brown), released into the cell cycle under the restrictive conditions. Data for WT parental cells (green) and *top2-td* cells (blue) from Figure 1 are shown for comparison. Graph shown is an average of two repeats. (C) The relative enrichment of H2AS129P over to H2A ChIP across centromeres is shown for *smc2-td K38I* cells (where Smc2 protein is depleted and an enzymatically inactive form of Smc2 smc2K38I is concurrently expressed; dark gray) and *smc2td K38I top2-td* cells where both smc2 and Top2 are depleted and *smc2K38I* expressed (orange), both released into the cell cycle under the restrictive conditions. Data for WT parental cells (green) and *top2-td* cells (blue) from Figure 1 are shown for comparison. Graph shown is generated from a pile up of the profiles of all centromeres and is an average of two repeats. (D) The relative enrichment of H2AS129P over H2A ChIP over the rDNA repeats is shown for *smc2-td K38I* cells (where Smc2 protein is depleted and an enzymatically inactive form of Smc2, smc2K38I, is concurrently expressed; dark gray) and *smc2-td K38I top2-td* cells where both smc2 and Top2 are depleted and *smc2K38I* expressed (orange), both released into the cell cycle under the restrictive conditions. Data for WT parental cells (green) and *top2-td* cells (blue) from Figure 1 are shown for comparison. Graph shown is an average of two repeats. See also Figures S3 and S7.

We next sought to identify why centromeric regions and the rDNA exhibit heightened DNA damage in response to topological stress during DNA replication. The SMC complex cohesin is loaded genome-wide onto the chromosomes from late G1 until anaphase but is primarily enriched at centromeres and the rDNA as cells enter S phase (Hu et al., 2015). Despite their large size, SMC complexes translocate along DNA (Lengronne et al., 2004, Terakawa et al., 2017), with DNA interaction regions found in several different parts of the complex (Baxter et al., 2019). Therefore, they could present a large yet mobile barrier to the diffusion of DNA topological stress around centromeres and across the rDNA by preventing free rotation of the DNA duplex. We assayed whether cohesin activity was required for the topologically linked DNA damage that accumulates across centromeres and over the rDNA. We used the well-characterized, temperature-sensitive *scc1-73* allele (Haering et al., 2004) to destabilize the cohesin complex in Top2-depleted cells during S phase. In both Top2-depleted cells and *top2-4* cells examined following completion of DNA replication, loss of cohesin activity completely suppressed the accumulation of H2AS129P across centromeric regions (Figures 4A and S3A). Over the rDNA, loss of cohesin suppressed H2AS129P to a similar level to that observed in unreplicated cells (Figures 4B and S3A).

The SMC complex condensin is also enriched at both the rDNA and centromeres in budding yeast (Wang et al., 2005). However, although rDNA condensin appears to be active from late S phase onward (Lavoie et al., 2004), centromeric condensin is not thought to be active until mitosis (Verzijlbergen et al., 2014). Cohesin and condensin activities at the rDNA are also linked with cohesin required for condensin-dependent compaction of the rDNA prior to anaphase (Lavoie et al., 2002). To examine whether condensin activity modulates levels of DNA damage following topological stress, we depleted both Top2 and the condensin subunit Smc2 and examined the extent of DNA damage detected on chromosomes following DNA replication. Across centromeres, we did not observe a condensin-dependent change in DNA damage in Top2-depleted cells (Figure 4C). In contrast, over the rDNA, condensin depletion suppressed the Top2-depletion-dependent increase in H2AS129P (Figure 4D). Condensin depletion also suppressed H2AP accumulation in cells with wild-type levels of Top2 (Figure 4D) to a similar extent as observed for cohesin disruption (Figure S3A). We conclude that both cohesin and condensin activities modulate the accumulation of DNA damage following topological stress, cohesin activity affects DNA-replication-associated DNA damage at centromeres, and both cohesin and condensin activities regulate levels of DNA damage across the rDNA array.

**Figure 5:**
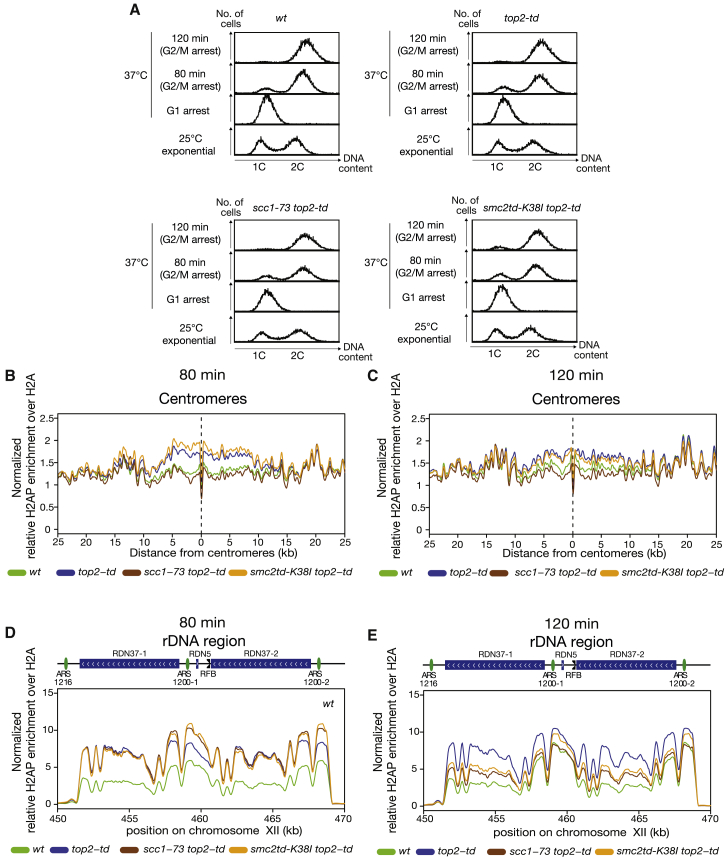
Quantitative ChIP-Seq of H2AS129P around Centromeres and rDNA Shows that Cohesin and Condensin Activity Affects Rate of Repair across the rDNA (A) FACS analysis of one repeat for wild type, *top2-td*, *scc1-73 top2-td*, or *smc2-td K38I top2-td* at 25°C exponential followed by restrictive conditions in a G1 arrest and released under restrictive conditions for both 80 min and 120 min. Second repeat is shown in Figure S7. (B) The relative enrichment of H2AS129P over H2A ChIP normalized to *S. pombe* spike in (see STAR Methods) around centromeres is shown for wild type (green), *top2-td* (blue), *scc1-73 top2-td* (brown), or *smc2-td K38I top2-td* (orange) all released into the cell cycle under the restrictive conditions for 80 min. Graph shown is generated from a pile up of the profiles of all centromeres and is an average of two repeats. (C) The relative enrichment of H2AS129P over H2A ChIP normalized to *S. pombe* spike in (see STAR Methods) around centromeres is shown for wild type (green), *top2-td* (blue), *scc1-73 top2-td* (brown), or *smc2-td K38I top2-td* (orange) all released into the cell cycle under the restrictive conditions for 120 min. Graph shown is generated from a pile up of the profiles of all centromeres and is an average of two repeats. (D) The relative enrichment of H2AS129P over H2A ChIP normalized to *S. pombe* spike in (see STAR Methods) across the rDNA repeats is shown for wild type (green), *top2-td* (blue), *scc1-73 top2-td* (brown), or *smc2-td K38I top2-td* (orange) all released into the cell cycle under the restrictive conditions for 80 min. Graph shown is an average of two repeats. (E) The relative enrichment of H2AS129P over H2A ChIP normalized to *S. pombe* spike in (see STAR Methods) across the rDNA repeats is shown for wild type (green), *top2-td* (blue), *scc1-73 top2-td* (brown), or *smc2-td K38I top2-td* (orange) all released into the cell cycle under the restrictive conditions for 120 min. Graph shown is an average of two repeats. See also Figure S4.

The loss of DNA damage at the centromeres and rDNA following disruption of cohesin or condensin activity could be either due to their activities causing DNA damage during DNA replication or their activity prolonging the presence of DNA damage by inhibiting DNA repair. To assess this, we examined cultures either 80 min after release from alpha factor, when cells were just completing DNA replication, or 120 min post-release, when all cells had completed DNA replication and held in the arrest state (Figure 5A). To ensure that analyses of the different states were quantitatively comparable, we spiked each of the crosslinked cultures with equal numbers of crosslinked *Schizosaccharomyces pombe* (*S. pombe*) cells where HO (homothallic switching) endonuclease had been activated to generate a constitutive double strand break (Watson et al., 2011), thus ensuring relatively high levels of H2AS129P in these cells (Figure S4A). We then used the *S. pombe* DNA immunoprecipitated with H2AS129P to normalize the genome-wide levels of H2AS129P in each of the tested budding yeast cultures. Notably, this process gave similar profiles for H2AS129P, both for *S. pombe* normalized and unnormalized *S. cerevisiae* ChIP-seq samples (Figures 5B–5E compared to Figures S4B and S4C). Normalized ChIP-seq experiments confirmed that depletion of Top2 led to the accumulation of H2AS129P at 80 min following release (Figure 5B). As expected, loss of cohesin function suppressed this accumulation around centromeres although loss of condensin did not (Figure 5B). After 120 min, we observed a lower level of H2AS129P in Top2-depleted samples (Figure 5C), consistent with the replicative lesions being repaired in the post-replicative arrest state. Decreased H2AS129P also occurred in condensin-depleted samples in 120 min relative to 80 min (Figures 5B and 5C), consistent with condensin not affecting DNA damage generation or repair around the centromeres. In cohesin-disrupted cells, similarly low levels of H2AS129P accumulated at 120 min as at 80 min (Figures 5B and 5C). We conclude that cohesin activity during DNA replication is required for generation of the DNA-topological-stress-dependent DNA damage at centromeres.

At the rDNA, we observed a distinct pattern of cohesin and condensin modulation of DNA damage. In the 80-min samples, we observed high levels of H2AS129P in all three Top2-depleted samples (Figure 5D), irrespective of whether cohesin or condensin activity was lost. In contrast, by 120 min after release from G1, DNA damage was still high across the rDNA in Top2-depleted samples but was now lower in cells where Top2 was depleted alongside disruption of cohesin or depletion of condensin (Figure 5E). Both cohesin and condensin are required for rDNA mitotic chromosome compaction at this arrest stage (Lavoie et al., 2002). We conclude that the mitotic chromosome structure generated by cohesin and condensin across the rDNA hinder the repair of the DNA-replication-dependent lesions generated by topological stress.

If cohesin-dependent topological stress is a significant cause of endogenous DNA replication stress at centromeres, we would predict that some cohesin-dependent DNA damage could still be detectable when Top2 is actively relaxing DNA topological stress. In post-replicative cells, H2AP levels across the rDNA were consistently higher in wild-type (WT) cells than *scc1-73* cells (Figure S3A), but no difference in the levels of H2AP were observed around centromeres between WT and *scc1-73* (Figure S3A). Previous analysis of the sites of endogenous DNA damage in budding yeast has indicated that H2AP accumulation around centromeres is limited to S phase (Szilard et al., 2010). To examine whether centromeric S phase DNA damage is dependent on cohesin, we compared H2AP by ChIP-seq in wild-type and *scc1-73* cells synchronized in G1, S (35 min post-release), and G2/M (Figure S3B). We observed that H2AP accumulates around centromeres in S phase cells, albeit in a less widespread manner than observed in Top2-depleted cells. Following disruption of cohesin (*scc1-73*), we observed lower levels of accumulation of H2AP in S phase cells around this region (Figure S3B), consistent with the presence of cohesin at centromeres stochastically disrupting DNA replication. However, FACS analysis of DNA content of our S phase samples indicates that *scc1-73* cells appeared to have advanced further through S phase than WT cells (Figure S3B). So we cannot exclude the possibility that this suppression may in part be related to the faster progress through S phase following disruption of cohesin.

**Figure 6:**
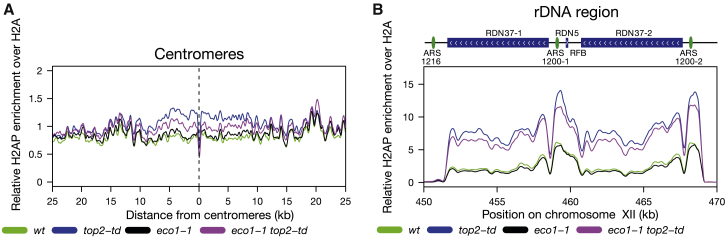
Inactivation of Eco1 Partially Suppresses H2AS129P Enrichment around Centromeres (A) The relative enrichment of H2AS129P over H2A ChIP around all centromeres in *eco1-1* cells either with WT expression of Top2 in *eco1-1* (black) or depleted of Top2 in *eco1-1 top2-td* (violet) cells, both released into the cell cycle under the restrictive conditions. Data for WT (green) and *top2-td* cells (blue) from Figure 1 are shown for comparison. Graph shown is generated from a pile up of the profiles of all centromeres and is an average of two repeats. (B) The relative enrichment of H2AS129P over H2A ChIP across the rDNA repeats in *eco1-1* cells either with WT expression of Top2 in *eco1-1* (black) or depleted of Top2 in *eco1-1 top2-td* (violet) cells, all released into the cell cycle under the restrictive conditions. Data for WT (green) and *top2-td* cells (blue) from Figure 1 are shown for comparison. Graph shown is an average of two repeats. See also Figure S7.

The acetylation of Smc3 by Eco1/Ctf7 stabilizes cohesin on DNA and promotes sister chromatid cohesion in *S. cerevisiae* (Rolef Ben-Shahar et al., 2008, Skibbens et al., 1999, Tóth et al., 1999, Unal et al., 2008). In human cells, Smc3 acetylation also regulates RF dynamics (Terret et al., 2009). To examine whether Eco1 activity affects the accumulation of DNA-topological-stress-linked DNA damage, we assayed cells depleted of both Top2 and the activity of the Eco1 acetyltransferase. Loss of Eco1/Ctf7 activity using the *eco1-1* allele partially suppressed H2AS129P accumulation across centromeres but did not substantially alter H2AS129P accumulation across the rDNA array of Top2-depleted cells (Figures 6A and 6B). Therefore, Smc3 acetylation is capable of modifying the extent of topological-stress-related DNA damage inflicted in cells but is not required for DNA damage per se.

**Figure 7:**
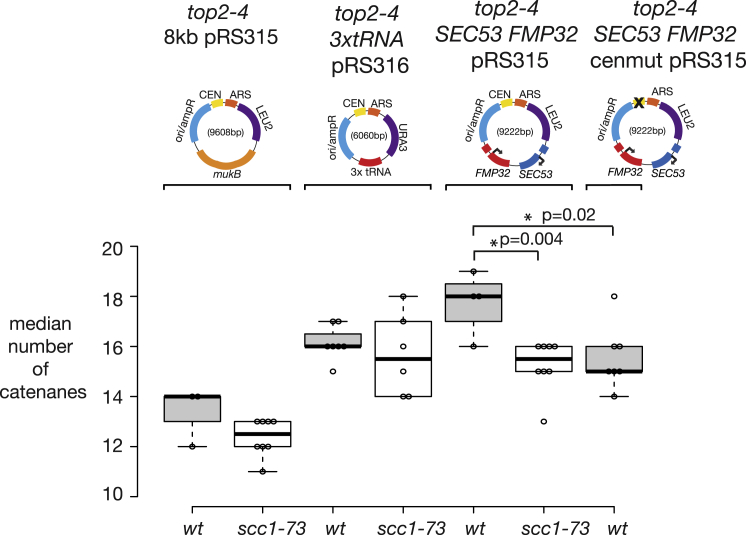
Cohesin Activity Increases Fork Rotation during DNA Replication of Plasmids with Active Transcription Units The frequency of fork rotation on the different plasmid replicons was examined by gel electrophoresis and Southern blotting as described in STAR Methods. Cells containing the *top2-4* allele and a CEN plasmid (8 kb *pRS315*), a CEN plasmid and 3× tRNA genes (3×*tRNA pRS316*), and a CEN plasmid with two active genes in a converging orientation (*SEC53 FMP32 pRS315*) were assessed for DNA catenation following one round of DNA replication in the absence of Top2 activity and with or without active cohesin (*WT* with active cohesin, *scc1-73* inactive cohesin). 3×*tRNA pRS316 WT* sample was taken from Schalbetter et al. (2015). The CEN plasmid with two active genes in a converging orientation, *SEC53 FMP32 pRS315*, was also modified to inactivate the centromere by a double point mutation (Jehn et al., 1991), *SEC53 FMP32 cenmutpRS315*, and assessed for DNA catenation following one round of DNA replication in the absence of Top2 activity. Representative autoradiograms of the *SEC53 FMP32 pRS315* plasmid with and without active cohesin and the *SEC53 FMP32 cenmutpRS315* plasmid are shown in Figure S6. The relative intensity of catenanes generated post-replication was quantified and the population median of the catenanes calculated for each of the conditions. The median of each experiment is plotted on the boxplot along with the boxes representing the middle two quartiles of the distributions of the dataset. p values are derived from paired t tests; a star indicates a significant difference between two conditions (p < 0.05). See also Figures S5 and S6.

Our data indicate that cohesin activity around centromeres leads to localized DNA topological stress during DNA replication. High levels of topological stress can drive RF reversal (Bermejo et al., 2011, Ray Chaudhuri et al., 2012, Postow et al., 2001b) or fork rotation, which relaxes topological stress ahead of the fork at the expense of generating pre-catenated intertwines behind the fork (Peter et al., 1998, Schalbetter et al., 2015). To directly test whether cohesin mediates RF dynamics through an accumulation of topological stress, we examined the effect of cohesin activity on the extent of fork rotation that occurs on DNA plasmids. Episomal plasmids that contain a point centromere load cohesin onto the DNA through the kinetochore. These DNA circles can be extracted from cells and probed for DNA-topology-dependent changes, including the extent of fork rotation through S phase, by agarose gel electrophoresis mobility (Schalbetter et al., 2015). An increase in fork rotation and pre-catenation can be directly observed if Top2 is prevented from resolving the pre-catenanes formed by fork rotation (Schalbetter et al., 2015). We tested whether loss of cohesin activity affected the extent of fork rotation and DNA pre-catenation on three different plasmids that are capable of loading cohesin: one containing a centromere; one containing a centromere and three different tRNA genes; and one containing a centromere and a pair of constitutively active converging genes. Loss of cohesin activity consistently reduced the extent of DNA catenation on the plasmid containing the converging gene pair, but not on either the centromeric plasmid alone or the centromeric plasmid containing tRNA genes (Figures 7 and S5A). We also found that ablating the primary cohesin binding site, by centromere inactivation on the plasmid with converging genes, also significantly reduced fork rotation (Figures 7 and S5B). These data indicate that the combination of chromatin-loaded cohesin and transcription-dependent DNA topological stress interferes with DNA replication, leading to elevated fork rotation on plasmids. Potentially, cohesin activity might cause this effect by directly altering the global extent of DNA supercoiling on the plasmid. However, we could observe no change in linking number on pre-replicative plasmids extracted from cells either with or without cohesin activity (Figure S6), indicating that cohesin activity did not introduce global DNA topological change into the plasmid but was rather trapping acute topological stress in the region ahead of the RF. We conclude that the loading and activity of cohesin in the context of active transcription lead to SMC complexes trapping increased acute DNA topological stress ahead of the DNA RF on these plasmids.

## Discussion

The SMC complexes have universal roles in maintaining chromosome stability in eukaryotes and repairing DNA lesions caused by replication stress. Here, we show another side to SMC activity, identifying distinct genomic contexts where cohesin activity disrupts DNA replication or delays the repair of lesions generated during S phase in budding yeast when DNA topological stress is not relaxed by Top2.

High levels of DNA topological stress are known to accumulate among the rDNA repeats due to 35S transcription (Schultz et al., 1992). Consistent with replication through such a DNA topologically stressed region resulting in a high frequency of fork rotation, the rDNA array is highly intertwined following DNA replication (D’Ambrosio et al., 2008). Surprisingly, the repair of the DNA lesions caused by DNA topological stress at the rDNA is hindered by both cohesin and condensin activity. In other contexts, SMC complexes promote repair of DNA damage (Sjögren and Nasmyth, 2001, Ström et al., 2007, Unal et al., 2007). We speculate that the known mitotic compaction functions of cohesin and condensin at the rDNA are responsible for impairing DNA repair across the array. This effect is potentially related to their function in inhibiting intra-chromatid chromosomal repair across the rDNA (Kobayashi and Ganley, 2005, Kobayashi et al., 2004).

In contrast, we find that the presence of the SMC complex cohesin around centromeric regions disrupts the normal course of DNA replication following topological stress, resulting in DNA damage and elevated levels of fork rotation. Across all centromeres, cohesin complexes are highly abundant, actively translocating to peri-centromeric regions from their loading site at the kinetochore (Hu et al., 2011, Hu et al., 2015, Petela et al., 2018). Establishment of sister chromatid cohesion across replicated centromeres is crucial for the bi-orientation of centromeres (Tanaka et al., 2000). Our data show that this essential activity comes at the cost of generating DNA topological stress around the centromere that has to be relaxed by topoisomerase action. If allowed to accumulate, it disrupts DNA replication, leading to DNA damage. In this model, loaded cohesin complexes would act as mobile topological barriers, trapping topological stress as they spread away from their loading site. Such a model initially appears to conflict with data showing that the passive movement of DNA-loaded cohesin complexes is not obviously constrained by stable DNA interaction (Ivanov and Nasmyth, 2007, Murayama and Uhlmann, 2015). However, SMC complexes have a range of different conformations, which are proposed to occur during active DNA translocation (Baxter et al., 2019, Çamdere et al., 2018, Hassler et al., 2018, Srinivasan et al., 2018). As SMC complexes pass through their enzymatic cycle, several DNA-binding domains become accessible at different enzymatic stages (Akai et al., 2011, Hirano and Hirano, 2006, Kschonsak et al., 2017). Indeed, *in vitro*, combining plasmid DNA with either condensin or cohesin can trap overwound DNA topology (Kimura and Hirano, 1997, Sun et al., 2013), a state only possible if the complex can distinguish distinct DNA topology via multiple DNA interactions during its enzymatic cycle. The trapping of DNA writhe by such a large complex would be predicted to make DNA-bound SMC complexes potent barriers to the diffusion of non-stabilized topological stress. The DNA topological stress that builds up adjacent to DNA-bound cohesin, particularly in regions where transcription provides a local source of topological stress, would then impede converging replication, especially when it is not rapidly resolved by topoisomerase II.

Because this model predicts that only the subset of SMC complexes that stabilize writhe would act as topological barriers, it follows that the effect on the RF would be most notable in regions with the highest density of SMC complexes in S phase. In budding yeast, this is across centromeric regions. Notably, in human cells, both cohesin complexes and Top2 accumulate at the bases of cohesin-dependent loops in interphase (Canela et al., 2017). These are also preferential sites of chromosome breakage in human cells (Canela et al., 2017). Thus, Top2 relaxation of cohesin-dependent DNA topological stress is also a feature of human cells.

How exactly the disruption of DNA replication by accumulated topological stress results in DNA damage is still unclear. Other studies on the third eukaryotic SMC complex SMC5/6 have suggested a link between the accumulation of DNA topological stress during DNA replication and the generation of DNA damage. SMC5/6 is enriched at sites of DNA damage and enriched following induction of topological stress in a cohesin-dependent manner (Lindroos et al., 2006, Jeppsson et al., 2014). Furthermore, SMC5/6 activity is associated with preventing endogenously generated DNA lesions developing into toxic recombination intermediates (Menolfi et al., 2015, Torres-Rosell et al., 2005). We speculate that the accumulation of SMC5/6 on chromosomes following DNA topological stress is in part due to its association with DNA lesions generated during DNA replication. Interestingly, the types of lesions thought to be acted on by SMC5/6, reversed forks (Xue et al., 2014) and single-stranded gaps behind the RF (Menolfi et al., 2015), are also those that have been proposed to be caused by DNA topological stress. Reversed forks are observed following induction of topological stress by Top1 poisoning (Ray Chaudhuri et al., 2012). Gapped, single-stranded sister chromatids are also proposed to arise following frequent fork rotation because frequent sister chromatid intertwining in the wake of the RF could inhibit replication processes occurring behind the fork, such as Okazaki fragment maturation (Schalbetter et al., 2015). Indeed, the entrapment of sister chromatids immediately behind the fork could exacerbate this effect.

We speculate that DNA topological stress due to SMC trapping must either be rapidly resolved by Top2 or locally regulated around RFs to ensure SMCs can efficiently carry out their conserved ability to act as a general protector of genome stability.

## STAR★Methods

### Key Resources Table

**Table:** 

REAGENT or RESOURCE	SOURCE	IDENTIFIER
**Antibodies**		

RPA1	Agrisera	Cat# AS07214; RRID: AB_1031803
H2A	Active Motif	Cat# 39235; RRID: AB_2687477
H2AS129P	Abcam	Cat# ab181447

**Chemicals, Peptides, and Recombinant Proteins**		

Bacto-yeast extract	Melford	Cat# Y1333
Bacto-peptone	Melford	Cat# P1328
Yeast Nitrogen Base	Melford	Cat# Y2004
Glucose	Sigma-Aldrich	Cat# G8270
Raffinose	Sigma-Aldrich	Cat# R0250
Galactose	Sigma-Aldrich	Cat# G0625
Adenine Sulfate	Formedium	Cat# DOC0230
L-Leucine	Formedium	Cat# DOC0157
L-Histidine	Formedium	Cat# DOC0145
Uracil	Formedium	Cat# DOC0214
Tris-HCl	Fisher Scientific	Cat# 10001223
Tris-base	Fisher Scientific	Cat# 10355910
EDTA	Fisher Scientific	Cat# 10716481
Boric Acid	Fisher Scientific	Cat# 10263370
Nb.BsmI	NEB	Cat# R0706
Nb.BsrDI	NEB	Cat# R0648
Megasieve Agarose	Flowgen	Cat# H15608
Hybond N+ membrane	GE Healthcare Amersham	Cat# RPN203B
Dextran Sulfate	Sigma-Aldrich	Cat# D8906
Tropix I-Block	Applied Biosystems	Cat# T2015
Flourescein tagged dUTP	Roche	Cat# 11373242910
Anti-fluorescein-AP Fab fragments	Roche	Cat# 11426338910
CDP-Star detection agent	GE Healthcare	Cat# RPN3682
Doxycycline (Dox)	Sigma-Aldrich	Cat# D9891
Alpha Factor	Genscript	CAS: 59401-28-4
Nocodazole (Noco)	Sigma-Aldrich	Cat# M1404
Propidium Iodide	Sigma-Aldrich	Cat# P4170
Pepsin	Sigma-Aldrich	Cat# P6887
Formaldehyde	Sigma-Aldrich	Cat# F8775
Phase Lock Gel Light 1.5 ml	Scientific Laboratory Supplies	Cat# 2302800
Glycine	Alfa Aesar	Cat# A13816
Protein A Dynabeads	Invitrogen	Cat# 10002D
Protein G Dynabeads	Invitrogen	Cat# 10004D
cOmplete, Mini, EDTA-free Protease Inhibitor Cocktail	Roche	Cat# 04693159001
PhosSTOP	Roche	Cat# PHOSS-RO
Yeast Synthetic Drop-out Medium Supplements without leucine	Sigma-Aldrich	Cat# Y1376
AMPure XP beads	Beckman Coulter	Cat# A63881
T4 DNA Polymerase	NEB	Cat# M0203
Yeast Synthetic Drop-out Medium Supplements without Uracil	Sigma-Aldrich	Cat# Y1501
DNase-free RNase	Roche	Cat# 11119915001
Sodium chloride (NaCl)	Fisher Scientific	Cat# 10428420
Sodium hydroxide (NaOH)	Fisher Scientific	Cat# 10254540
Trisodium citrate	Fisher Scientific	Cat# 10448610
Hydrochloric acid (HCl)	Fisher Scientific	Cat# 10316380
Ethanol absolute	Sigma-Aldrich	Cat# E7023
Lyticase	Sigma-Aldrich	Cat# L2524
Phenol	Sigma-Alrdich	Cat# P4557
Chloroform	Fisher Scientific	Cat# 10293850
isoamylalcohol	Fisher Scientific	Cat# 10786661
Chloroquine	Sigma-Aldrich	Cat# C6628
β-mercaptoethanol	Sigma-Aldrich	Cat# 63689
Proteinase K	Invitrogen	Cat# 10124532
Tween 20	Sigma-Aldrich	Cat# P1379
Triton X-100	Sigma-Aldrich	Cat# T9284
Sodium dodecyl sulfate (SDS)	Fisher Scientific	Cat# 10090490
RNaseA	Sigma-Aldrich	Cat# R4875

**Critical Commercial Assays**		

NEBNext Ultra II library kit	NEB	Cat# E7645
NEBNext Multiplex Oligos for Illumina sets 1-4	NEB	Cat# E7335, E7500, E7710, E7730
QIAGEN PCR purification kit	QIAGEN	Cat# 28106
QuikChange Lightning Site-Directed Mutagenesis Kit	Agilent Technologies	Cat# 210518

**Deposited Data**		

Processed sequencing data	This paper	GSE131558
Raw Southern blots	This paper	https://dx.doi.org/10.17632/hx92v9jtm2.1

**Experimental Models: Organisms/Strains**		

*S. cerevisiae* W303 background. *Mat a ade2-1 his3-11 leu2-3 trp1-1 ura3-1 can1-100*	Baxter laboratory	Baxter lab strain 1
*S. cerevisiae* W303 background. *Mat a ade2-1 his3-11 leu2-3 trp1-1 ura3-1 can1-100 UBR1::GAL1-10-Ubiquitin-M-LacI*	Tanaka and Diffley, 2002	Baxter lab strain 12
*fragment-Myc-UBR1 (HIS3) leu2-3::pCM244 (CMVp-tetR’-SSN6, LEU2) x3 pRS316*		
*S. cerevisiae* W303 background. *Mat a ade2-1 his3-11 leu2-3 trp1-1 ura3-1 can1-100 UBR1::GAL1-10-Ubiquitin-M-LacI*	This paper	Baxter lab strain 1991
*fragment-Myc-UBR1 (HIS3) leu2-3::pCM244 (CMVp-tetR’-SSN6, LEU2) x3*		
*S. cerevisiae* W303 background. *Mat a ade2-1 his3-11 leu2-3 trp1-1 ura3-1 can1-100 UBR1::GAL1-10-Ubiquitin-M-LacI*	This paper	Baxter lab strain 13
*fragment-Myc-UBR1 (HIS3) leu2-3::pCM244 (CMVp-tetR’-SSN6, LEU2) x3 top2-td TOP2 5′ upstream −100 to −1 replaced with kanMX-tTA (tetR-VP16)-tetO2 - Ub - DHFRL80P-ts - Myc –linker) pRS316*		
*S. cerevisiae* W303 background. *Mat a ade2-1 his3-11 leu2-3 trp1-1 ura3-1 can1-100 UBR1::GAL1-10-Ubiquitin-M-LacI*	This paper	Baxter lab strain 1992
*fragment-Myc-UBR1 (HIS3) leu2-3::pCM244 (CMVp-tetR’-SSN6, LEU2) x3 top2-td TOP2 5′ upstream −100 to −1 replaced with kanMX-tTA (tetR-VP16)-tetO2 - Ub -DHFRL80P-ts - Myc –linker)*		
*S. cerevisiae* W303 background. *Mat a ade2-1 his3-11 leu2-3 trp1-1 ura3-1 can1-100 UBR1::GAL1-10-Ubiquitin-M-LacI*	This paper	Baxter lab strain 171
*fragment-Myc-UBR1 (HIS3) leu2-3::pCM244 (CMVp-tetR’-SSN6, LEU2) x3 top2-td TOP2 5′ upstream −100 to −1 replaced with kanMX-tTA (tetR-VP16)-tetO2 - Ub - DHFRL80P-ts - Myc -linker scc1-73 pRS316*		
*S. cerevisiae* W303 background. *Mat a ade2-1 his3-11 leu2-3 ura3-1 can1-100 scc1-73 trp1Δ:: hphNT1*	McAleenan et al., 2012	Baxter lab strain 211
*S. cerevisiae* W303 background. *Mat a ade2-1 his3-11 leu2-3 trp1-1 ura3-1 can1-100 UBR1::GAL1-10-Ubiquitin-M-LacI*	This paper, derived from Tercero et al., 2000	Baxter lab strain 2083
*fragment-Myc-UBR1 (HIS3) leu2-3::pCM244 (CMVp-tetR’-SSN6, LEU2) x3 CDC45::cdc45-td (CUP1p-Ub-DHFRts-HA-CDC45)(TRP1)*		
*S. cerevisiae* W303 background. *Mat a ade2-1 his3-11 leu2-3 trp1-1 ura3-1 can1-100 UBR1::GAL1-10-Ubiquitin-M-LacI*	This paper	Baxter lab strain 2085
*fragment-Myc-UBR1 (HIS3) leu2-3::pCM244 (CMVp-tetR’-SSN6, LEU2) x3 CDC45::cdc45-td (CUP1p-Ub-DHFRts-HA-CDC45)(TRP1) top2-td TOP2 5′ upstream −100 to −1 replaced with kanMX-tTA (tetR-VP16)-tetO2 - Ub - DHFRL80P-ts - Myc –linker)*		
*S. cerevisiae* W303 background. *Mat a his4-539 ura3-52 top2-4*	Holm et al., 1985	Baxter lab strain 479
*S. cerevisiae* W303 background. *Mat a his4-539 lys2-801 ura3-52 top2-4 pRS316-3x tRNA*	Schalbetter et al., 2015	Baxter lab strain 484
*S. cerevisiae* W303 background. *Mat a ade2-1 his3-11 leu2-3 trp1-1 ura3-1 can1-100 UBR1::GAL1-10-Ubiquitin-M-LacI*	This paper	Baxter lab strain 500
*fragment-Myc-UBR1 (HIS3) leu2-3::pCM244 (CMVp-tetR’-SSN6, LEU2) x3 trp1-1::SDM-pFA6a-GAL1-SMC2-K39I-6HA, TRP1 smc2-td SMC2 5′ upstream-100 to −1 replaced with kanMX-tTA (tetR-VP16) -tetO2 -Ub -DHFRts −3xHA extended linker)*		
*S. cerevisiae* W303 background. *Mat a ade2-1 his3-11 leu2-3 trp1-1 ura3-1 can1-100 UBR1::GAL1-10-Ubiquitin-M-LacI*	This paper	Baxter lab strain 2122
*fragment-Myc-UBR1 (HIS3) leu2-3::pCM244 (CMVp-tetR’-SSN6, LEU2) x3 trp1-1::SDM-pFA6a-GAL1-SMC2-K39I-6HA, TRP1 smc2-td SMC2 5′ upstream-100 to −1 replaced with kanMX-tTA (tetR-VP16) -tetO2 -Ub -DHFRts −3xHA extended linker) top2-td TOP2 5′ upstream −100 to −1 replaced with kanMX-tTA (tetR-VP16)-tetO2 - Ub - DHFRL80P-ts - Myc -linker)*		
*S. cerevisiae* W303 background. *Mat a ade2-1 leu2-3 his4-539/his3-11 ura3-52/ura3-1 can1-100 top2-4*	This paper	Baxter lab strain 1307
*scc1-73 trp1Δ:: hphNT1*		
*S. cerevisiae* W303 background. *Mat a ade2-1 his4-539/his3-11 lys2-801 ura3-52/ura3-1 can1-100 top2-4 scc1-73 trp1Δ::hphNT1 leu2Δ::natNT2*	This paper	Baxter lab strain 1313
*SEC53 FMP32 pRS315 (converging)*		
*S. cerevisiae* W303 background. *Mat a ade2-1 his4-539/his3-11 lys2-801 ura3-52/ura3-1 can1-100 top2-4 scc1-73 trp1Δ::hphNT1 leu2Δ::natNT2*	This paper	Baxter lab strain 1315
*mukB pRS315 (genes removed using bglII)*		
*S. cerevisiae* W303 background. *Mat a ade2-1 his4-539/his3-11 lys2-801 ura3-52/ura3-1 can1-100 top2-4 scc1-73 trp1Δ::hphNT1 leu2Δ::natNT2*	This paper	Baxter lab strain 1317
*pRS316-3tRNA*		
*S. cerevisiae* W303 background. *Mat a ade2-1 his3-11 leu2-3,112 trp1-1 ura3-1 can1-100 top2-4 mukB pRS315 (genes removed using bglII)*	This paper	Baxter lab strain 1323
*S. cerevisiae* W303 background. *Mat a ade2-1 his3-11 leu2-3 trp1-1 ura3-1 can1-100 UBR1::GAL1-10-Ubiquitin-M-LacI*	This paper	Baxter lab strain 1412
*fragment-Myc-UBR1 (HIS3) leu2-3::pCM244 (CMVp-tetR’-SSN6, LEU2) x3 eco1-1 (G211H)*		
*S. cerevisiae* W303 background. *Mat a ade2-1 his3-11 leu2-3,112 trp1-1ura3-1 can1-100 top2-4 SEC53 FMP32* pRS315 *cen-mut* (converging)	This paper	Baxter lab strain 1455
*S. cerevisiae* W303 background. *Mata ade2-1 his3-11 leu2-3 trp1-1 ura3-1 can1-100 UBR1::GAL1-10-Ubiquitin-M-LacI*	This paper	Baxter lab strain 1489
*fragment-Myc-UBR1 (HIS3) leu2-3::pCM244 (CMVp-tetR’-SSN6, LEU2) x3 top2-td TOP2 5′ upstream −100 to −1 replaced with kanMX-tTA (tetR-VP16)-tetO2 - Ub - DHFRL80P-ts - Myc -linker) eco1-1 (G211H)*		
*S. cerevisiae* W303 background. *Mat a his4-539 ura3-52*	This paper	Baxter lab strain 1496
*top2-4 SEC53 FMP32 pRS315* (converging)		
*Schizosaccharomyces pombe h− urg1::Purg1lox-HO, LEU-HOcs-his3+-λ-EU2, leu1-32, his3-D1*	Watson et al., 2011	AW507

**Oligonucleotides**		

Cenmut_F1: AAGAAATTAAAGAAAAAATAGTTTTTG TTTTCATAAGATGTAAAAGACTCTAGGGGGATCG	This Paper	N/A
Cenmut_R1: CGATCCCCCTAGAGTCTTTTACATC TTATGAAAACAAAAACTATTTTTTCTTTAATTTCTT	This paper	N/A

**Software and Algorithms**		

Illumina Basespace	N/A	http://basespace.illumina.com/auth/logon?returnUrl=https%3A%2F%2Fbasespace.illumina.com%2Fhome%2Findex
Bowtie2	Langmead and Salzberg, 2012	http://bowtie-bio.sourceforge.net/bowtie2/index.shtml
Samtools	Li et al., 2009	http://samtools.sourceforge.net/
Model-based Analysis of ChIP-Seq (MACS2)	Zhang et al., 2008	https://github.com/taoliu/MACS
R Programme version 1.1.447	R core team	https://www.R-project.org/

### Lead Contact and Materials Availability

Further information and requests for resources and reagents should be directed to and will be fulfilled by the lead contact Jon Baxter Jon.Baxter@sussex.ac.uk.

All unique/stable reagents generated in this study are available from the Lead Contact without restriction.

### Experimental Model and Subject Details

#### Yeast Strains

Yeast containing *top2-td* were derived from W303-1a (*MATa ade2-1 ura3-1 his3-11, trp1-1 leu2-3, can1-100*), *top2-4* cells derived from Holm et al. (1985), backcrossed onto the W303-1 background and grown at 25°C. Full genotypes are listed in the Key Resources Table. For the spike-in normalization experiment AW507 *S. pombe* strain (Watson et al., 2011) was grown at 30°C.

### Method Details

#### Plasmid Generation

Plasmid centromere point mutation in *SEC53 FMP32 cenmutpRS315* plasmid was created using QuikChange Lightning Site-Directed Mutagenesis Kit (Agilent technologies). A double point mutation was inserted into the CDE III region of CEN 6 based on Jehn et al. (1991) at bp 4-A and bp 5-T.

#### Media and Cell Cycle Synchronization

*top2-td* cell cultures for alpha factor release experiments were prepared as described previously (Schalbetter et al., 2015): cultures were grown in YP media with 40 mg/l adenine + 2% raffinose to midlog phase. Cells were then arrested in G1 with 10 μg/ml alpha factor (Genscript) until 90% of cells were in G1 (120 min). 2% galactose and 20 minutes later 50 μg/ml doxycycline (Sigma-Aldrich) was added. 30 minutes after galactose addition cultures were incubated at 37°C for 1h and cells were released from the block into YP + 40 mg/l adenine + 2% raffinose + 2% galactose. Time 0 was taken as time of addition of the first wash. Nocodozole (Sigma-Aldrich) was added to cultures at 10 μg/ml 45 minutes after 0. Samples were then fixed for ChIP-SEQ analysis at indicated time points.

For plasmid experiments with *top2-4* strains, yeast cells were grown in Yeast Nitrogen Base + yeast synthetic drop-out medium (Sigma-Aldrich) + 2% glucose, selecting for the plasmid (-ura or -leu) to log phase at 25°C, before transferring to YP + 40 mg/l adenine + 2% glucose and grown to midlog phase. Cells were then arrested in G1 with 10 μg/ml alpha factor until 90% of cells were in G1 (120 minutes). The culture was incubated at 37°C for 1h and cells were released from the block into YP + 40 mg/l adenine + 2% glucose. Time 0 was taken as time of addition of first wash. Nocodozole (Sigma-Aldrich) was added to cultures at 10 μg/ml. Samples were taken at the indicated time points, pelleted and frozen on dry ice.

For the spike-in experiments cells were grown as described previously (Watson et al., 2011): primary cultures of *S. pombe* cells were grown in 100 mL EMM media supplemented with + 100 μg/ml leucine on 30°C to log phase and re-inoculated in 1 l EMM + 100 μg/ml leu to grow overnight to reach ∼5 × 10^6^ cell concentration. Cells were then re-suspended in pre-warmed EMM supplemented with 100 μg/ml leucine, histidine and uracil to induce endonuclease production. Cells were then incubated for 2h before cell fixation.

#### Flow cytometry (FACS) analysis

FACS analysis was carried out as in Schalbetter et al. (2015): 500 μl of culture samples were re-suspended in 70% ethanol for fixing. They were then spun down and re-suspended in 1 mL of 50 mM Tris-HCl pH8 with 5 mg/ml RNaseA (Sigma-Aldrich) at 37°C overnight. Samples were pelleted and re-suspended in 5 mg/ml pepsin (Sigma-Aldrich) and 5 μl/ml concentrated HCl. They were then incubated at 37°C for 30 minutes. Samples were pelleted and washed in 50 mM Tris-HCl pH8 before being re-suspended in 50 mM Tris-HCl pH8 with 0.5 mg/ml Propidium iodide (Sigma-Aldrich) and sonicated ready for analyzing using the BD Accuri C6 sampler and analyzed using FCS express 4 flow software. FACS analysis for all the ChIP-SEQ experiments are shown in Figure S7.

#### Fixation for ChIP-SEQ library preparation

Cultures were fixed at 25°C in YP + 1% formaldehyde (Sigma-Aldrich) for 45 minutes. 125 mM glycine (Alfa Aesar) was then added for 5 minutes. Cells were washed with PBS before being pelleted and frozen in liquid nitrogen. *S. pombe* cells for spike-in experiments were fixed the same way, and after PBS wash were resuspended in 10 mL cold PBS and aliquoted to 250 ul stocks, before being pelleted and frozen in liquid nitrogen.

#### ChIP-SEQ

Pellets from 50 mL culture were resuspended in 500 μL SDS buffer (1% SDS, 10 mM EDTA, 5M Tris HCl, cOmplete Tablets, Mini EDTA-free EASYpack (Roche), PhosSTOP (Roche)). Cells were lysed in a FASTPREP machine, 5 rounds of 1 min at 6.5 power, with 200 μL of 0.5 mm silica beads. Lysate was spun out and IP buffer (0.1% SDS, 1.1% Triton X-100, 1.2 mM EDTA, 16.7 mM TRIS HCl (pH8), cOmplete Tablets, Mini EDTA-free EASYpack (Roche), PhosSTOP (Roche)) was added to a final volume of 1 ml. Samples were sonicated using the Focused-Ultrasonicator (Covaris, M220) (Average incident power – 7.5 Watts, Peak Incident Power – 75 Watts, Duty Factor – 10 %, Cycles/Burst – 200, Duration – 20 min). The sample was centrifuged for 20 min at 13,000 rpm at 4°C. Supernatant was then diluted to 1:10 (5 mL total). 50 μL protein A Dynabeads (Invitrogen) and 50 μL protein G Dynabeads (Invitrogen), were washed 3 times in IP buffer followed by adding to the sample and incubating for 2 h at 4°C. Supernatant was split, with 2X 2 mL being taken to 15 mL Falcon tubes, and 1 mL being kept at −20°C as an input sample. To the two 2 mL samples antibody was added, either H2A 1:500 (active motif) or 1.6 μg/ml H2AP (Abcam), and these were placed on a rotating wheel at 4°C for 15 – 20 h. For experiments where RPA1 ChIP was performed on the same sample, 75 mL starting cultures were used meaning supernatant could be split into 3X 2ml and 1 mL for input, with RPA1 antibody (1:10000, Agrisera) added to one 2 mL aliquot. For experiments where RPA1 ChIP was exclusively performed, 25 mL starting cultures were used to make a split of 1X 2ml for antibody addition and 1ml for input.

A preparation of Dynabeads (Invitrogen), Protein A (30 μl) and Protein G (30 μl), was washed 3 times in IP buffer. This was added to each sample and incubated at 4°C for 4 h. Supernatant was removed and beads were washed at 4°C for 6 min in TSE-150 (1% Triton X-100, 0.1% SDS, 2 mM EDTA, 20 mM Tris HCl (pH8), 150 mM NaCl), followed by TSE-500 (1% Triton X-100, 0.1% SDS, 2 mM EDTA, 20 mM Tris HCl (pH8), 500 mM NaCl), followed by LiCl wash (0.25 M LiCl, 1% NP-40, 1% dioxycholate, 1 mM EDTA, 10 mM Tris HCl (pH8)) and finally Tris-EDTA (TE pH8). Elution was carried out in 400 μL elution buffer, for 30 min at room temperature. At the same time 50 μL from the input sample was added to 150 μL of elution buffer. 20 μL of 5 M NaCl and 10 μL of 10 mg/ml proteinase K (Invitrogen) was then added to the input, and 40 μL and 20 μL to the IP samples respectively. These were incubated at 65°C overnight. Then 10 μL of DNase-free RNase (Roche) was added to the input and 20 μL to the IP samples, and they were left at 37°C for 30 min. All DNA was purified with a QIAGEN PCR purification kit and eluted in 50 μL for H2A or H2AP or 40 μL for RPA1. DNA amount was measured using the Qubit 2.0 Fluorometer (Life technologies) as per the manufacturer’s instructions.

For H2A or H2AP samples, libraries were prepared using the NEBnext Ultra II library kit (NEB) as per the manufacturer’s instructions. PCR enrichment required 13 cycles. PCR purification was carried out using AMPure XP beads. For RPA1 library preparation 34 ul from the RPA1 samples and 1 ng DNA in 34 ul water from the input were used. 5 μl 10 x NEB2.1 buffer and 5 μl of random primers (8N, 3 mg/ml stock) were added and the samples were boiled at 95°C for 5 minutes and immediately placed to ice for 5 minutes. 5 μl 10 x dNTP with dUTP instead of dTTP (2 mM each) and 1 μl T4 polymerase (NEB) were added and the mixture was incubated at 37°C in a thermal cycler for 20 min, and 5 μl 0.5 M EDTA (pH 8) was immediately added to stop the reaction. The resulting dsDNA was used to create libraries using the Ultra II library kit (NEB) as per the manufacturer’s instructions. Paired end sequencing was performed using the MySeq (75bp reads from each side) or NextSeq 500 (42 bp reads from each side) systems.

For spike-in experiments aliquots of cell pellets was resuspended in 250 μl SDS buffer, and 1/1000 volume of the original *S. cerevisiae* culture (corresponding to 1:10 *S. pombe* to *S. cerevisiae* ratio) was added to each *S. cerevisiae* samples which were then processed the same way as described above.

#### DNA preparation for gel electrophoresis

Frozen pellets were re-suspended in lysis buffer (50mM Tris-HCl pH 8.0, 100mM NaCl, 10mM EDTA, 1%SDS) and the cell wall removed by incubation with 80 units/ml Lyticase (Sigma-Aldrich) and 1% β-mercaptoethanol (Sigma-Aldrich) at 37°C for 5 minutes. DNA was then extracted with phenol/chloroform/isoamylalcohol (25:24:1) and the aqueous layer removed using phase lock tubes (Scientific Laboratory Supplies). DNA was precipitated with 2 volumes of 100% ethanol and washed with 70 % ethanol before being re-solubilized in 10mM Tris pH8.0.

#### Gel electrophoresis for plasmid catenation

For catenation 2D gels the DNA was nicked with either Nb.BsmI or Nb.BsrDI (NEB) according to the manufacturer’s instructions. Nicked catenanes were separated in the first dimension on a 0.4% agarose (Megasieve, Flowgen) gel in 1x TBE (Tris-base, Boric Acid, EDTA) at 1.2V/cm for 13-17h at room temperature. The respective lanes were excised and embedded into a 0.8%–1.2% (depending on plasmid size) agarose (Megasieve, Flowgen) gel and run at 2-4.8V/cm in 1x TBE (at 4°C if more than 2V/cm were used).

#### Gel electrophoresis for plasmid supercoiling

DNA was separated in the first dimension on a 0.4% agarose (Megasieve, Flowgen) gel in 1X TBE + 0.5 μg/ml chloroquine (Sigma-Aldrich). Running conditions were 1.2V/cm for 20h at room temperature, in the dark. The gel was incubated in 1X TBE + 1 μg/ml chloroquine for 3 h. The respective lanes were excised and embedded into a 1.2% agarose (Megasieve, Flowgen) gel + 1 μg/ml chloroquine and run at 4.8V/cm in 1x TBE at 4°C for 10h.

#### Southern blotting

Non-radioactive Southern blotting and detection were carried out as described in Baxter et al. (2011): the gel was washed in depurination buffer (0.125 M HCl), denaturation buffer (0.5 M NaOH, 1.5 M NaCl) and neutralization buffer (0.5 M Tris-HCl, 1.5 M NaCl pH 7.5). DNA was transferred onto Hybond-N+ membrane (GE Healthcare) by capillary action in 20X SCC (NaCl, Trisodium citrate, pH 7). After transfer, DNA was ultraviolet cross-linked to the membrane using a UV Stratalinker 1800 (Stratagen) at 1200 J/m. The membrane was blocked at 60°C (5X SSC, 5% Dextran Sulfate (Sigma-Aldrich), 0.2% Tropix I-Block (Applied Biosystems), 0.1% SDS). Plasmid DNA was probed with DNA amplified from sequences of pRS315 or pRS316. Labeling and detection used random prime labeling module incorporating fluorescein tagged dUTP (Roche). Washes were carried out at 60°C in 1X SSC with 0.1% SDS, followed by 0.5X SSC and 0.1% SDS. The membrane was blocked in AB buffer (100 mM Tris-HCl, 150 mM NaCl (pH 7.5)) with 1% milk. Hybridized fluorescein tagged dUTP was detected with alkaline phosphatase Anti-fluorescein-AP Fab fragments (Roche) followed by washing in AB buffer + 0.2% Tween 20 (Sigma-Aldrich) and revealed with CDP-Star (GE Healthcare). Non-saturating exposures acquired on an ImageQuant LAS4000 system (GE Healthcare). Densitometry analysis was carried out using ImageQuant TL software. Overexposed images were taken to clearly identify the CatAn = 1 signal, which was often weak in non-saturating exposures.

### Quantification and Statistical Analysis

#### ChIP-SEQ analysis

##### FASTQ files were generated by Illumina basespace

(http://basespace.illumina.com/auth/logon?returnUrl=https%3A%2F%2Fbasespace.illumina.com%2Fhome%2Findex). The resulting sequences were aligned to a reference genome (R64-1-1, *Saccharomyces cerevisiae* S288c assembly from *Saccharomyces* Genome Database) using Bowtie 2 generating a SAM output file for each sample (http://bowtie-bio.sourceforge.net/bowtie2/index.shtml). Reads from MiSeq were trimmed 25 bp from 3′ and 1 bp from the 5′ end, while reads from NextSeq were not trimmed.

##### Command for MiSeq reads

bowtie2 -p 14 -x [path to index folder]–trim3 25–trim5 1 −1 [Path and name of R1 fastq file] −2 [Path and name of R2 fastq file] -S [name of the resulting .sam file]

##### Command for NextSeq reads

bowtie2 -p 14 -x [path to index folder] –trim3 0–trim5 0 −1 [Path and name of R1 fastq file] −2 [Path and name of R2 fastq file] -S [name of the resulting .sam file]

##### SAM files were then converted into sorted BAM files by using SAMtools (http://samtools.sourceforge.net/)

samtools sort [name of the .sam file generated with bowtie2] -o [name for the resulting .bam file] -O bam -T [name for temporary file (optional, used if parallel nodes are used)]

##### For RPA1 analysis duplicates were then removed using picard (https://broadinstitute.github.io/picard)

java -jar ∼/picard/picard-tools-1.138/picard.jar MarkDuplicates I = [name for the resulting .bam file] O = [name for the resulting without repeats.bam file] M = [name of metrix file.txt] REMOVE_DUPLICATES = true

BAM files were used for Model-based Analysis of ChIP-SEQ (MACS2). We used the ‘call peak’ function which also generates genome wide score data. These were used to generate fold enrichment tracks. Example command:

macs2 callpeak -t [sorted BAM file from yh2a data]-c [sorted BAM file from h2a data]-f BAMPE -g 12100000 -n [name for output file] -B -q 0.01–SPMR

The data then was sorted into 50 bp bins, normalized to have a mean value of 1, smoothed by a moving average of 7 bins, and used for meta data analysis using custom made R programs.

For spike-in experiments only reads that uniquely aligned to either *S. pombe* or *S. cerevisiae* genome were used from input, H2A and H2AP samples. To extract uniquely aligned reads for *S. cerevisiae* fastq files were first aligned to *S. pombe* genome (Downloaded from https://www.pombase.org/downloads/genome-datasets (9/4/2018)), then the unaligned read reads were aligned to *S. cerevisiae*. To get unique reads for *S. pombe* genome, fastq files were first aligned to *S. cerevisiae* and the unaligned reads were then aligned to *S. pombe* genome.

##### Example command lines for obtaining unique reads for *S. cerevisiae*

Getting unaligned reads from sam file generated by aligning fastq reads to *S. pombe* genome:

samtools view -b -F2 [name of the .sam file generated with bowtie2] > [name for the resulting .bam file]

Generating sorted .bam files from the unmapped reads:

samtools sort -n [name for the .bam file from previous step] -o [name for the resulting sorted .bam file] -O bam -T [name for temporary file (optional, used if parallel nodes are used)]

Output .fastq files from unaligned reads for subsequent alignment to *S. cerevisiae* genome using bedtools:

bedtools bamtofastq -i [name for the sorted .bam file] -fq [name for the resulting fastq file R1 reads] -fq2 [name for the resulting fastq file R2 reads]

The resulting .fastq files were then aligned to *S. cerevisiae.* Reads uniquely aligned to *S. pombe* were obtained similarly and the data were processed the same way as before to generate enrichment tracks over input or H2A. *S. cerevisiae* H2AP enrichment tracks were then normalized to *S. pombe* enrichment using custom R scripts, based on the normalization method used in Hu et al. (2015) where they used the equation:

Wc∗IPx/Wx∗IPc=OR

Where W_c_ = whole cell extract (input) counts from control genome (*S. pombe* in our case), IP_x_ = IP counts from experimental genome (*S. cerevisiae*), W_x_ = whole cell extract (input) counts from experimental genome (*S. cerevisiae*), IPc = IP counts from control genome (*S. pombe*) and OR = occupancy ratio – the normalizing factor used to multiply count numbers of each experimental sample.

However, rather than raw counts we used H2AP enrichment over input data from the MACS2 analysis which accounts for local biases and background noise as well as providing better representation of the repetitive sequences. As H2AP enrichment is basically calculated by IP counts/input counts reorganizing the equation above gives:

OR = Enrichment in experimental genome/enrichment in control genome. Based on this, H2A enrichment over input per bin was calculated for *S. cerevisiae* (C_b_) and for *S. pombe* (P_b_) by dividing the sum of all H2AP enrichment in every bin over input by the number of bins in the genome. Occupancy ratio (OR – the normalizing factor) was then calculated by dividing C_b_ by P_b_. OR was then used to multiply H2AP enrichment values over H2A for *S. cerevisiae* to generate normalized relative enrichment values. These values were then smoothed by a moving average of 7 bins.

### Data and Code Availability

The accession number for the processed sequencing data reported in this paper is GEO: GSE131558.

The accession number for the raw Southern blots reported in this paper is Mendeley Data: https://dx.doi.org/10.17632/hx92v9jtm2.1

## Supplemental Information

Document S1. Figures S1–S7

Document S2. Article plus Supplemental Information
