# Supplementary material for: Cohesin Causes Replicative DNA Damage by Trapping DNA Topological Stress
Source: Mol Cell. 2020 May 21;78(4):739–751.e8. doi: 10.1016/j.molcel.2020.03.013 (PMC7242899; doi:10.1016/j.molcel.2020.03.013)
Supplement: Document S1. Figures S1–S7 [file mmc1.pdf]

Molecular Cell, Volume *78*

Supplemental Information

Cohesin Causes Replicative DNA Damage by Trapping DNA Topological Stress

Nicola Elizabeth Minchell, Andrea Keszthelyi, and Jonathan Baxter

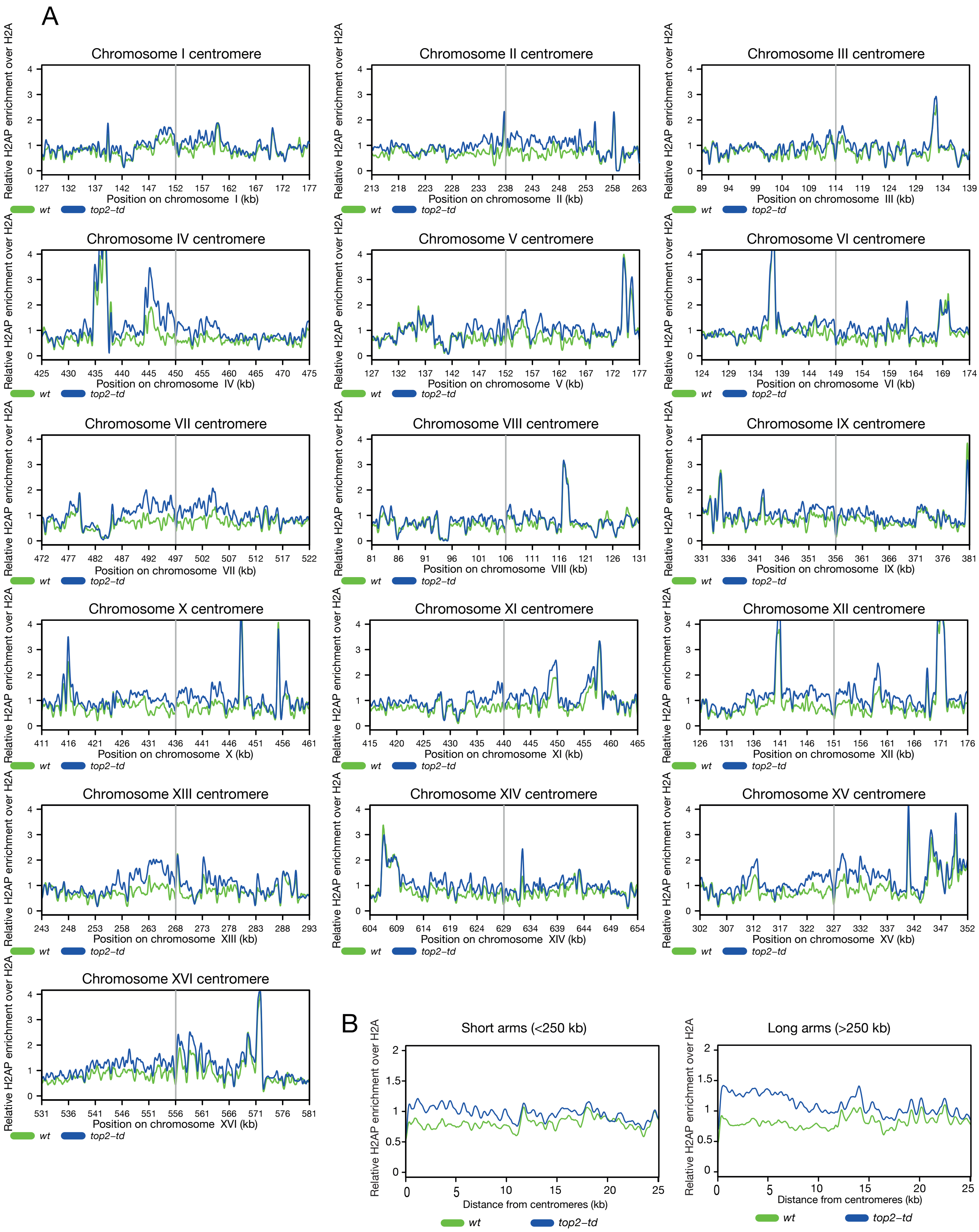

Figure S1, related to Figure 1. Depletion of Top2 during S phase causes H2AS129P enrichment at centromeres.

(A) Relative enrichment of H2AS129P over H2A ChIP around each individual centromere, averaged for two repeats, for either *wt* (green) or *top2-td* cells (blue), both released into the cell cycle under the restrictive conditions. Graph shown is an average of two repeats using data from Figure 1.
(B) Relative enrichment of H2AS129P over H2A ChIP at centromeres at short (<250 kb) and long arms (>250 kb), for either *wt* (green) or *top2-td* cells (blue), both released into the cell cycle under the restrictive conditions (data from Figure 1). Plots were generated by piling enrichment data starting from the centromeres up to 25 kb distance on each chromosome arm, and is an average of two repeats.

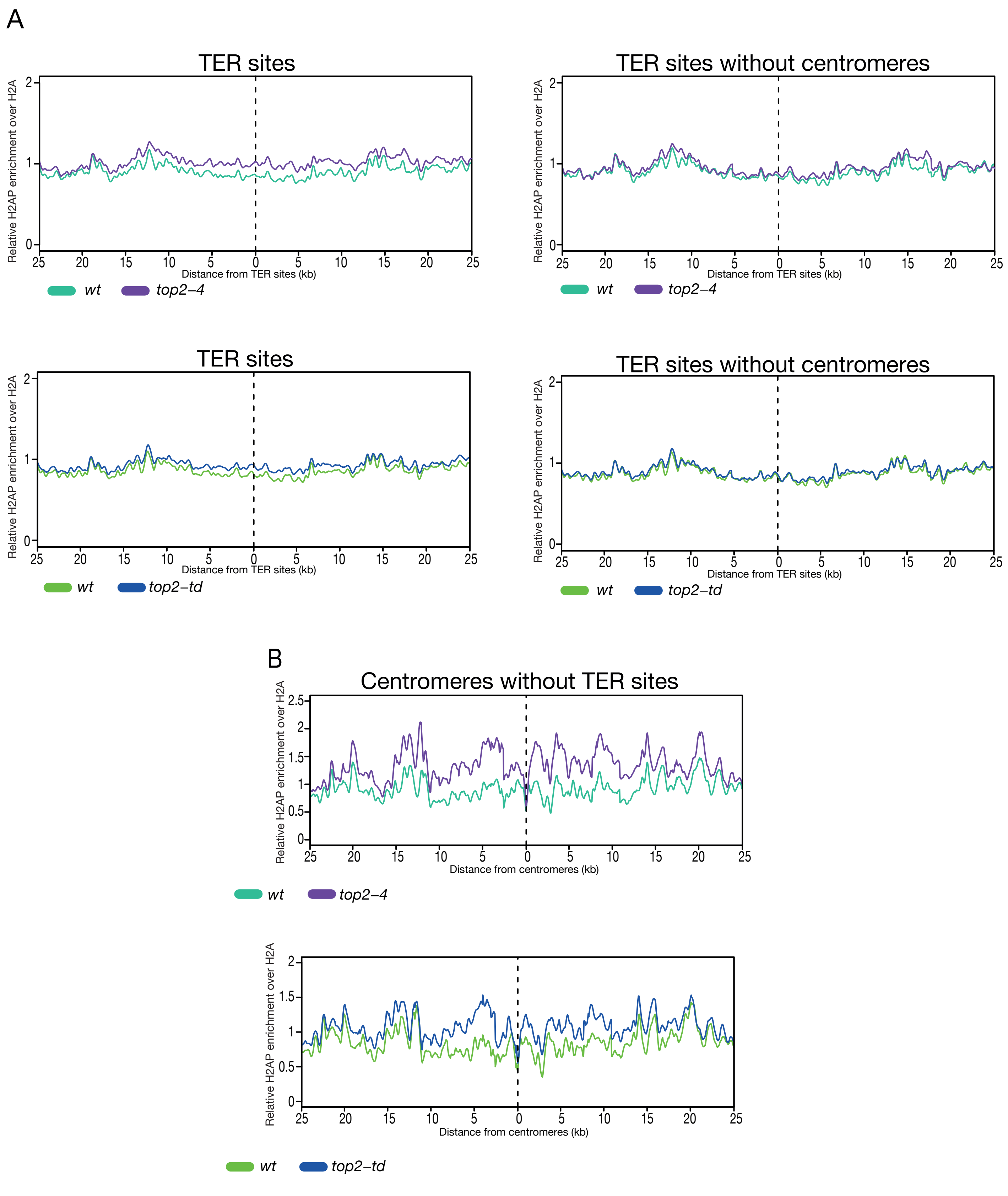

Figure S2, related to Figure 1. H2AS129P enrichment at centromeres caused by depletion of Top2 during S phase is independent of TER sites.

(A) The relative enrichment of H2AS129P over H2A ChIP around TER sites (defined by Fachinetti et al., 2010) (left panels) or around TER sites where data 10 kb either side of centromeres was removed (right panels). *wt* (turqoise) or *top2-4* (purple) (top panels) and *wt* (green) or *top2-td* cells (blue) (lower panels), all released into the cell cycle under the restrictive conditions. Graph shown is an average of two repeats using data from Figure 1.

(B) The relative enrichment of H2AS129P over H2A ChIP around centromeres, where data 10 kb either side of any TER sites was removed. *wt* (turquoise) or *top2-4* (purple) (top panel) and *wt* (green) or *top2-td* cells (blue) (lower panel), all released into the cell cycle under the restrictive conditions. Graph shown is an average of two repeats using data from Figure 1.

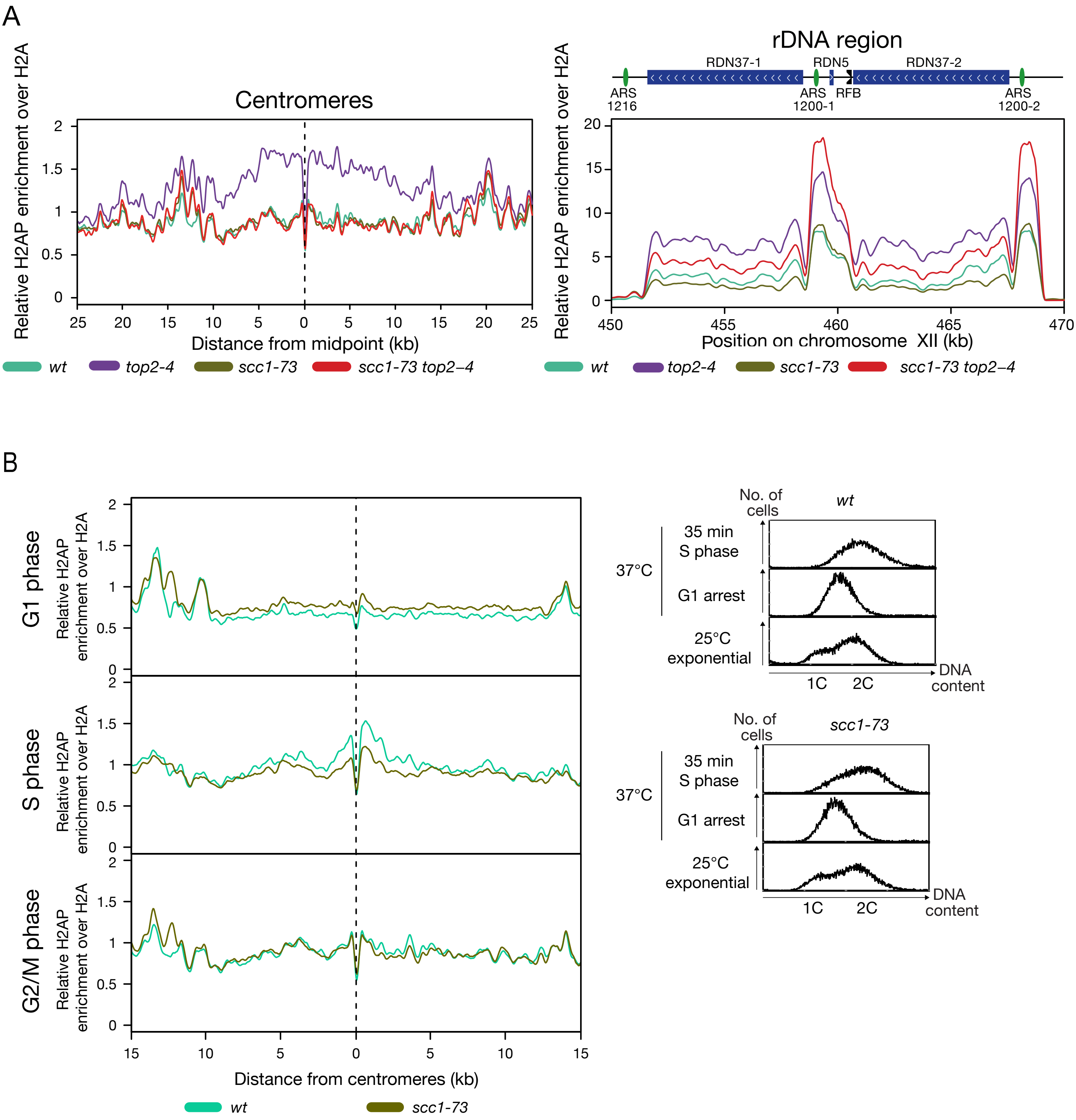

Figure S3, related to Figure 4. DNA damage at centromeres and rDNA is dependent on cohesin.
(A) The relative enrichment of H2AS129P over H2A ChIP across centromeres (left panel) or across the rDNA repeats (right panel) is shown for *scc1-73* (olive green) or *scc1-73 top2-4* (red), both released into the cell cycle under the restrictive conditions. Data for *wt* (turquoise) and *top2-4* (purple) from Figure 1 is shown for comparison. Left panel graph is generated from a pile up of the profiles of all centromeres. Both graphs shown are an average of two repeats. (B) The relative enrichment of H2AS129P over H2A ChIP (left panel) in *wt* (turquoise) and *scc1-73* (olive green) cells under restrictive conditions in G1 phase (top panel), and released into S phase under restrictive conditions for 35 minutes (middle panel) and in G2/M phase (lower panel – data used from Figure S3A). Graph shown is generated from a pile up of the profiles of all centromeres and is an average of two repeats. Right panel shows FACS analysis of one repeat of DNA content of each of the G1 and S samples examined by ChIP-SEQ. Second repeat shown in Figure S7.

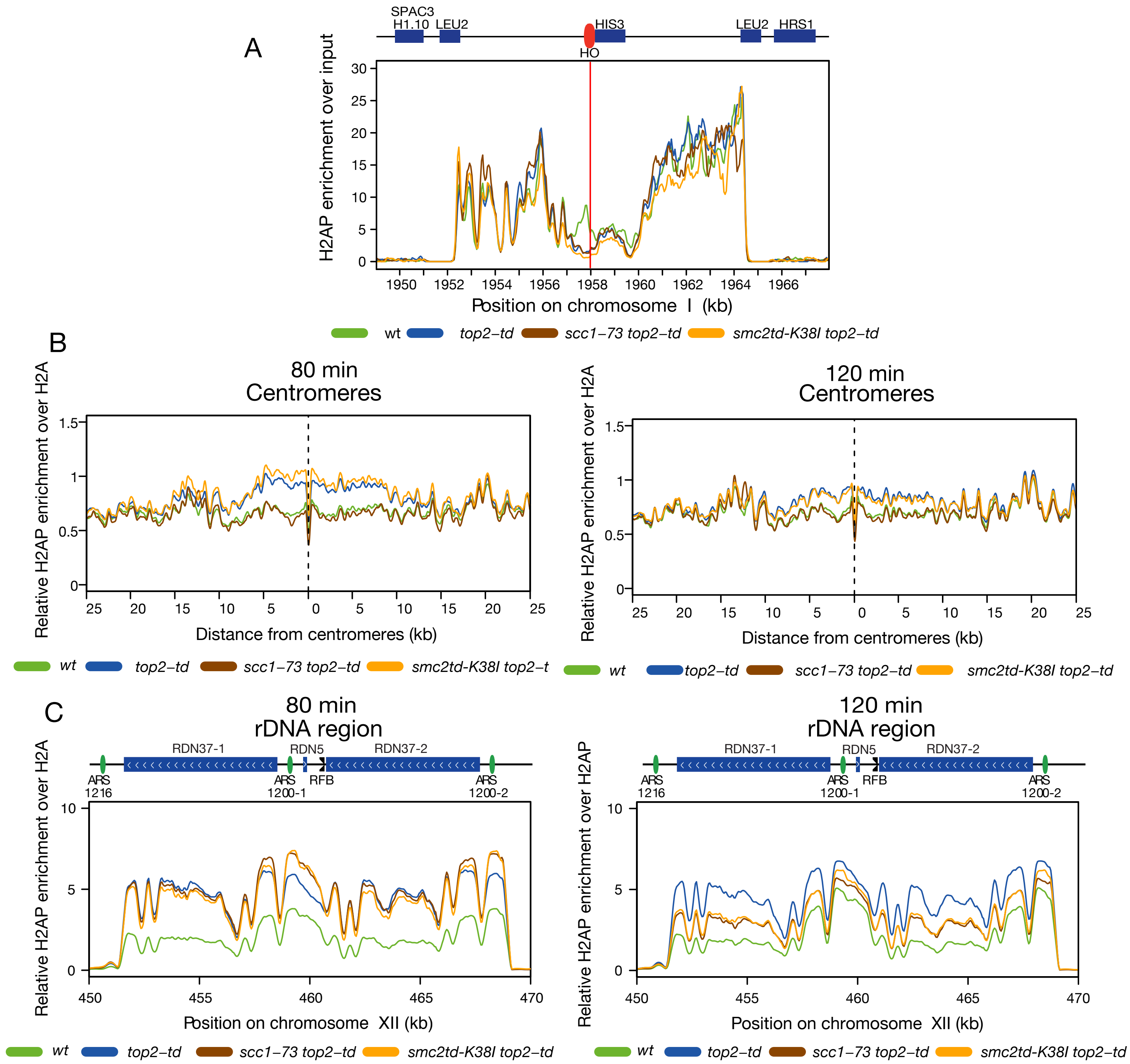

Figure S4, related to Figure 5. *S. pombe* normalized and unnormalized *S. cerevisiae* ChIP-SEQ analysis show similar profiles for H2AS129P enrichment.
(A) H2AP enrichment over input in *S. pombe* around the HO site (described in Watson et al., 2011) after induction of the endonuclease from samples mixed with S. cerevisiae *wt* (green), *top2-td* (blue), *scc1-73 top2-td* (brown), or *smc2-td K38I top2-td* (orange) 80 min after release into restrictive conditions from alpha factor (data used is one repeat from Figure 5).
(B) The relative enrichment of H2AS129P over H2A ChIP without the S. pombe spike normalization process around centromeres is shown for wildtype *wt* (green), *top2-td* (blue), *scc1-73 top2-td* (brown), or *smc2-td K38I top2-td* (orange) all released into the cell cycle under the restrictive conditions for 80 minutes (left panel) or 120 minutes (right panel). Graphs shown use data from Figure 5, they are generated from a pile up of the profiles of all centromeres and are an average of two repeats.
(C) The relative enrichment of H2AS129P over H2A ChIP without the S. pombe spike normalization process across the rDNA repeats is shown for wildtype *wt* (green), *top2-td* (blue), *scc1-73 top2-td* (brown), or *smc2-td K38I top2-td* (orange) all released into the cell cycle under the restrictive conditions for 80 minutes (left panel) or 120 minutes (right panel). Graphs shown use data from Figure 5 and are an average of two repeats.

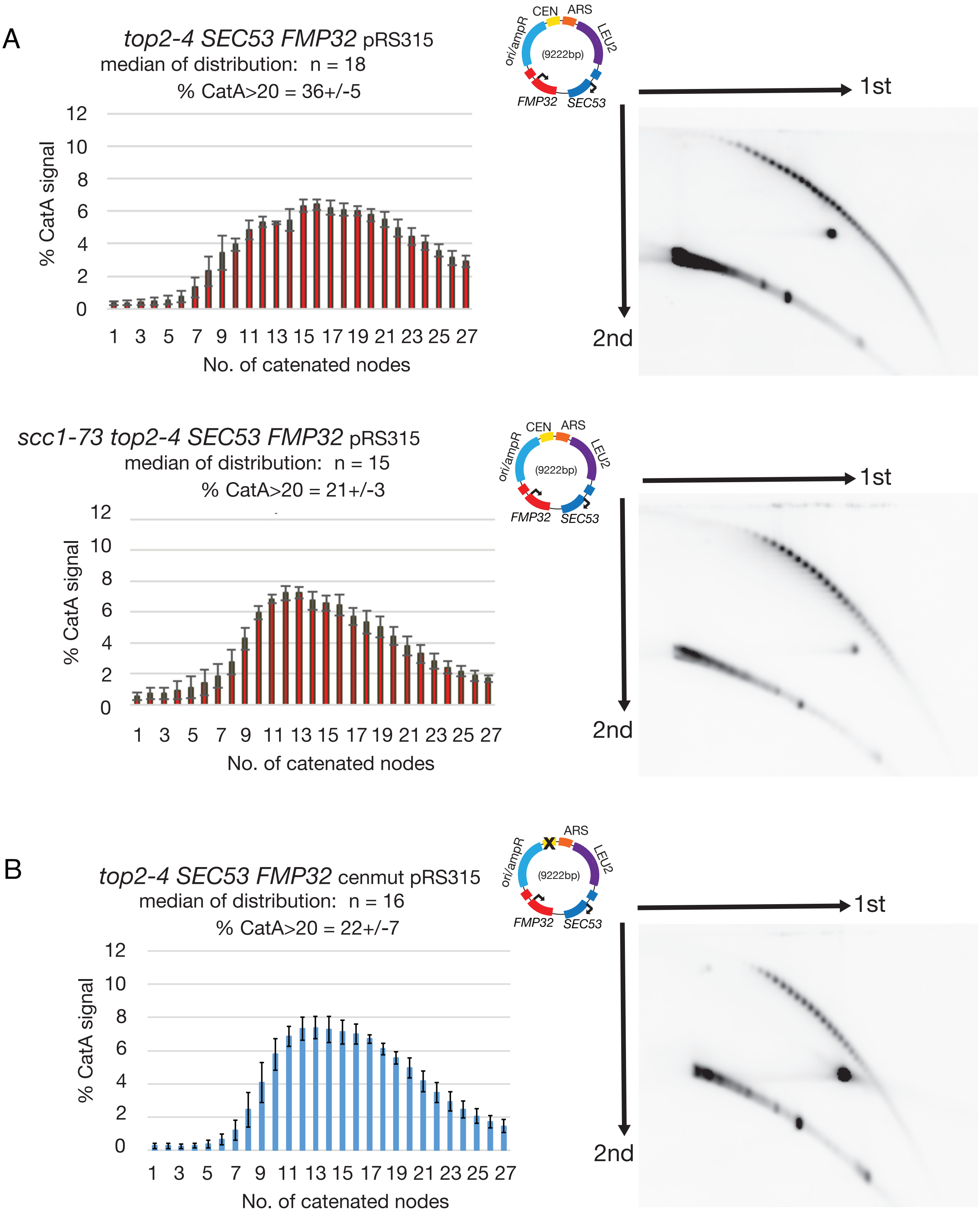

Figure S5, related to Figure 7. Cohesin and cohesin loading sites are required for fork rotation on the converging genes plasmid during DNA replication.

(A) The frequency of fork rotation in *top2-4 SEC53 FMP32 pRS315* (top panel) and *scc1-73 top2-4 SEC53 FMP32 pRS315* (bottom panel) cells was assessed in S phase by analysing DNA catenation on the plasmid following one round of DNA replication in the absence of Top2 activity. The relative intensity of catenanes generated post replication was quantified and the population median of the catenanes calculated for each of the conditions calculated. Representative autoradiograms are shown. Histograms showing the relative distribution of the intensity of catenanes generated post replication were quantified and calculated from 4 individual experiments (top panel) and 8 individual experiments (bottom panel) with the median of the averages and % of catenanes over 20 indicated. Error bars represent the average deviation of the repeats. Medians from each individual experiment are shown in Figure 7.

(B) The frequency of fork rotation in *SEC53 FMP32 cenmutpRS315* was examined as above. Representative autoradiograms are shown. Histograms showing the relative distribution of the intensity of catenanes generated post replication was quantified and calculated from 7 individual experiments with average median and % of catenanes over 20 indicated. Medians from each individual experiment are shown in Figure 7.

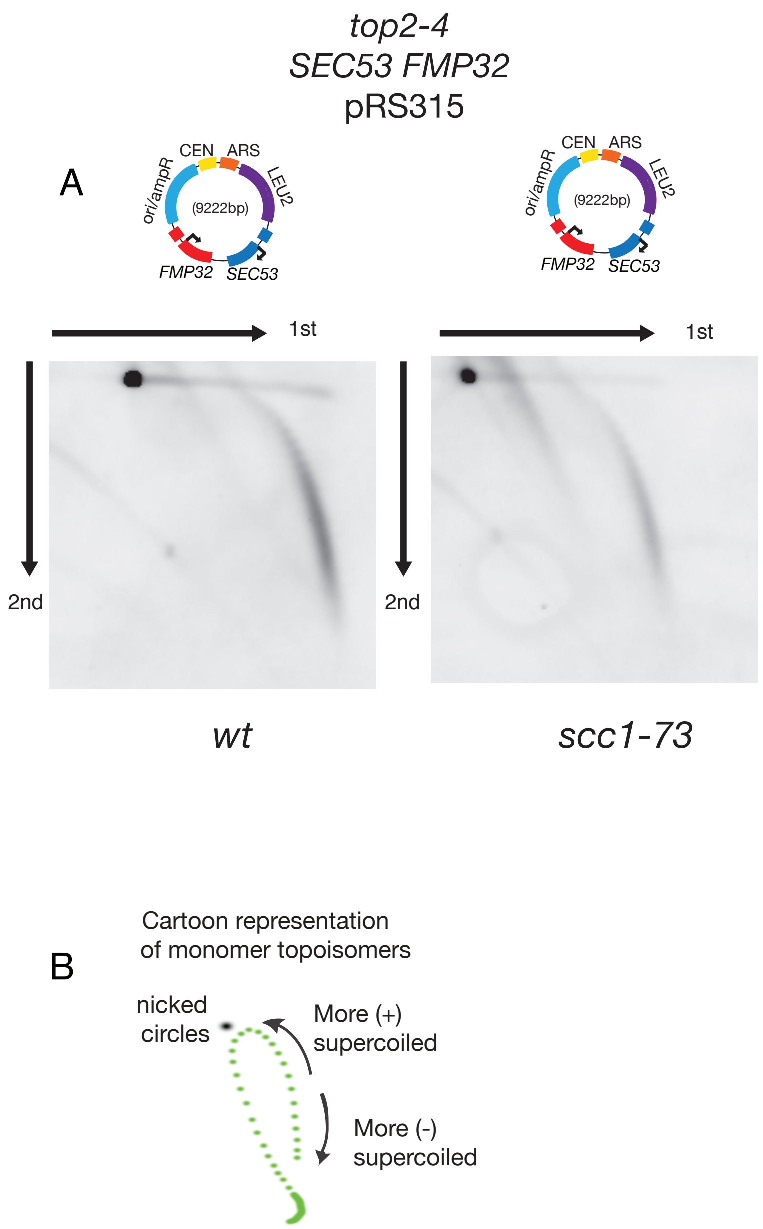

Figure S6, related to Figure 7. Cohesin activity does not change the global linking number of plasmids prior to DNA replication.
(A) Both *top2-4 SEC53 FMP32 pRS315* (left panel) and *scc1-73 top2-4 SEC53 FMP32 pRS315* (right panel) were synchronously arrested in G1 with alpha factor and then released into S phase for 30 min in the restrictive conditions to allow entry into S phase whilst also ensuring that most plasmid remains unreplicated and monomeric and therefore suitable for DNA supercoiling analysis. Purified DNA was analysed in 2D chloroquine gels (see STAR methods) to reveal the supercoiling distribution of the monomer plasmids. One of three repeats is shown for (left panel) and one of four repeats for (right panel).
(B) A cartoon representation of how the plasmid distribution relates to supercoiling status is shown.

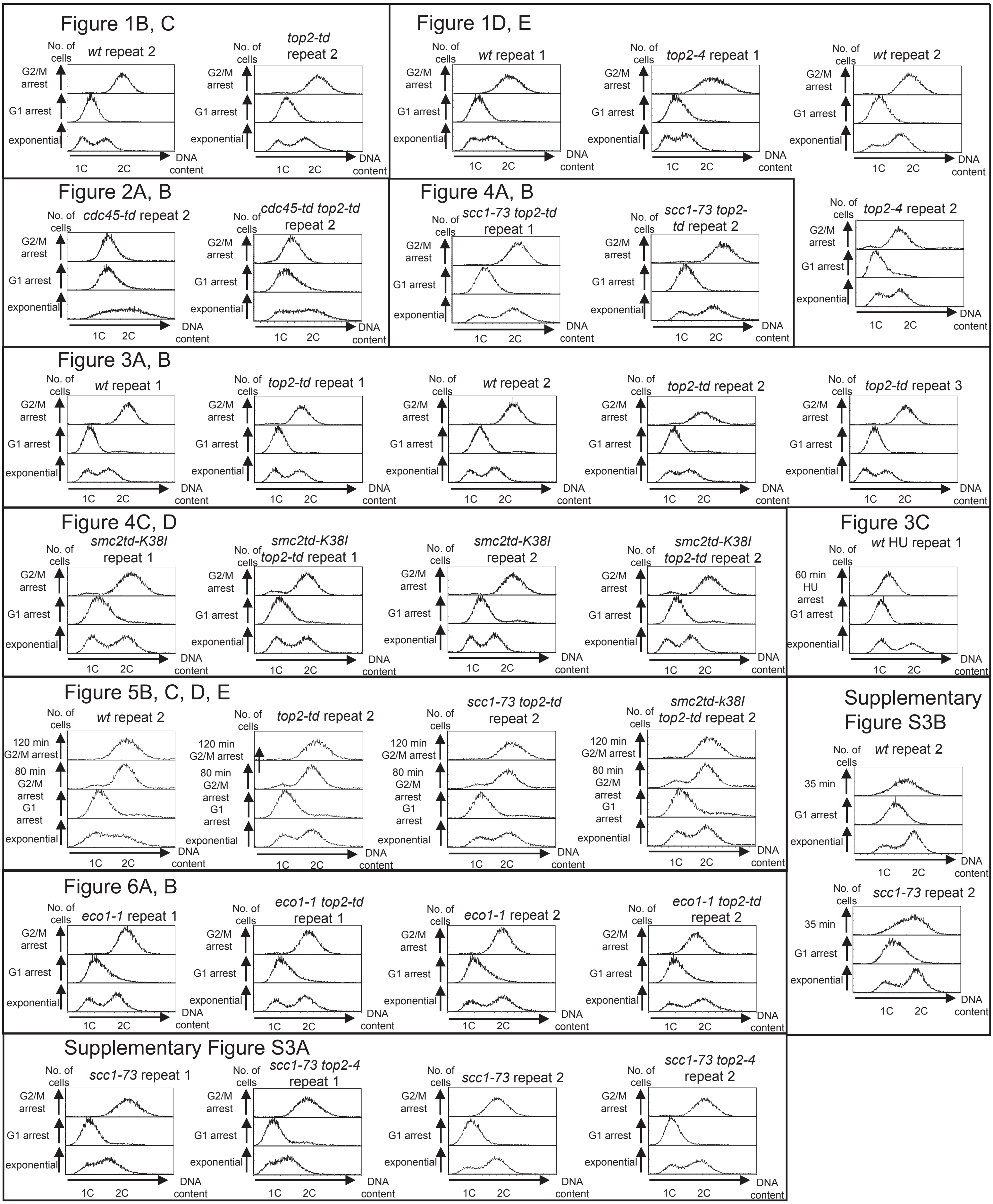

Figure S7, Related to STAR Methods. FACS analysis of all the experiments used for ChIP SEQ.

Figures indicated represent the first use of each sample in the paper. Samples from Figure 1B, C were also used in Figure 2A, B, Figure 4A-D, Figure 6A, B, Figure S1A, B and Figure S2A, B. *wt* from Figure 1B, C was also used to make a RPA1 library in Figure 3A, B. The first repeat for Figure 1B, C is shown in Figure 2C. Samples from Figure 1D, E were also used in Figure S2A, B and Figure S3A, B. Samples from Figure 5B, D were also used in Figure S4A-C. Samples from Figure 5C, E were also used in Figure S4B, C. The first repeat for Figure 5B-E is shown in Figure 5A. The first repeat for Figure S3B G1 and S phase samples are shown in Figure S3B.
